# Establishment and characterization of a novel vincristine‐resistant diffuse large B‐cell lymphoma cell line containing the 8q24 homogeneously staining region

**DOI:** 10.1002/2211-5463.12538

**Published:** 2018-11-20

**Authors:** Shohei Mizuno, Ichiro Hanamura, Akinobu Ota, Sivasundaram Karnan, Jo Kanasugi, Ayano Nakamura, Souichi Takasugi, Kaori Uchino, Tomohiro Horio, Mineaki Goto, Satsuki Murakami, Mayuko Gotou, Hidesuke Yamamoto, Masaya Watarai, Masato Shikami, Yoshitaka Hosokawa, Hiroshi Miwa, Masafumi Taniwaki, Ryuzo Ueda, Masakazu Nitta, Akiyoshi Takami

**Affiliations:** ^1^ Division of Hematology Department of Internal Medicine Aichi Medical University Japan; ^2^ Department of Biochemistry Aichi Medical University Japan; ^3^ Department of Hematology Daiyukai General Hospital Aichi Japan; ^4^ Department of Hematology Mie University Japan; ^5^ Department of Hematology and Oncology Graduate School of Medical Science Kyoto Prefectural University of Medicine Japan; ^6^ Department of Tumor Immunology Aichi Medical University School of Medicine Japan

**Keywords:** chromosome 8q24, diffuse large B‐cell lymphoma, homogeneously staining region, *MYC*, oncogene amplification, patient‐derived cell line

## Abstract

Chromosome band 8q24 is the most frequently amplified locus in various types of cancers. *MYC* has been identified as the primary oncogene at the 8q24 locus, whereas a long noncoding gene, *PVT1*, which lies adjacent to *MYC*, has recently emerged as another potential oncogenic regulator at this position. In this study, we established and characterized a novel cell line, AMU‐ML2, from a patient with diffuse large B‐cell lymphoma (DLBCL), displaying homogeneously staining regions at the 8q24 locus. Fluorescence *in situ* hybridization clearly detected an elevation in *MYC* copy numbers corresponding to the homogenously staining region. In addition, a comparative genomic hybridization analysis using high‐resolution arrays revealed that the 8q24 amplicon size was 1.4 Mb, containing the entire *MYC* and *PVT1* regions. We also demonstrated a loss of heterozygosity for *TP53* at 17p13 in conjunction with a *TP53* frameshift mutation. Notably, AMU‐ML2 cells exhibited resistance to vincristine, and cell proliferation was markedly inhibited by *MYC*‐shRNA‐mediated knockdown. Furthermore, genes involved in cyclin D, mTOR, and Ras signaling were downregulated following *MYC* knockdown, suggesting that *MYC* expression was closely associated with tumor cell growth. In conclusion, AMU‐ML2 cells are uniquely characterized by homogenously staining regions at the 8q24 locus, thus providing useful insights into the pathogenesis of DLBCL with 8q24 abnormalities.

**Abbreviations**
aCGH: array comparative genomic hybridization
CCND1: cyclin D1
CNA: copy number alteration
DLBCL: diffuse large B‐cell lymphoma
FISH: fluorescence *in situ* hybridization
GSEA: gene set enrichment analysis
HSR: homogeneously staining region
PBL: peripheral blood leukocyte
PVT1: plasmacytoma variant translocation 1
qRT‐PCR: quantitative reverse transcription‐polymerase chain reaction
R‐CHOP: rituximab plus cyclophosphamide, doxorubicin, vincristine, and prednisolone
R‐Hyper‐CVAD/MA: high‐dose methotrexate and cytarabine

Gene amplification, observed in the form of double‐minute chromosomes or homogeneously staining regions (HSRs), is recurrent and plays an important role in cancer 1. HSR is rarely seen in hematopoietic neoplasms compared with solid tumors and is observed at a lower frequency in lymphoid neoplasms than in myeloid neoplasms 2.

Chromosome 8q24 is the most frequently amplified locus in many cancers, with *MYC* being the most likely oncogene at this locus. The *MYC* gene encodes a transcription factor that regulates the expression of many target genes that control cell proliferation. The deregulation of *MYC*, resulting from t(8;14) and gene amplification, leads to the constitutive overexpression of *MYC* in numerous cancers and plays a pathogenetic role in oncogenesis 3, 4.

Plasmacytoma variant translocation 1 (*PVT1*) is also located at 8q24, 57 kb downstream of *MYC,* and extends over 200 kb in the direction of the telomeres. *PVT1* is a non‐protein‐coding gene and a homologue of mouse *Pvt1* 5. The *Pvt1* locus is a site of recurrent translocation in mouse plasmacytomas and a common integration site for the murine leukemia virus, which is capable of inducing T‐cell lymphomas in mice. In contrast to the typical Burkitt lymphoma (BL), in which the t(8;14) translocation contains a breakpoint within *MYC*, the t(2;8) or t(8;22) variant translocations in BL contain breakpoints in *PVT1* 6.

*PVT1* produces a variety of noncoding RNAs, including several microRNAs 7. The precise functions of the *PVT1* region and its noncoding RNAs remain unclear, although the long noncoding *PVT1* RNA has a documented role in stabilization of the MYC protein 8. Moreover, several groups have reported that the amplification and subsequent overexpression of *PVT1* have an oncogenic function in ovarian and breast cancers 9. A genomewide screen using array comparative genomic hybridization (array‐CGH) and gene expression profiling identified *PVT1* as a candidate oncogene in breast and ovarian cancers, acute myeloid leukemia, and Hodgkin lymphoma 10, 11. Furthermore, we have previously reported two novel chimeric genes, *PVT1‐NBEA* and *PVT1‐WWOX,* in multiple myeloma cell lines 12.

In addition, a circular RNA obtained from exon 3 of *PVT1* (*circPVT1*) has been shown to function as a promoter of cell proliferation in fibroblasts 13, an important prognostic factor in gastric cancer 14, and a diagnostic marker in osteosarcoma 15, and to have an oncogenic role in head and neck carcinoma and myeloid leukemia 16, 17. Hu *et al*. 18 have also described *circPVT1* overexpression in B‐cell acute lymphoblastic leukemia and performed functional studies indicating its role in B‐cell proliferation. Taken together, these reports highlight the potential importance of the *MYC*/*PVT1* locus in the pathophysiology of many cancers.

Diffuse large B‐cell lymphoma (DLBCL) is the most common type of non‐Hodgkin lymphoma and is known as a biologically heterogeneous tumor. Although approximately 70% of patients with DLBCL survive longer than five years when treated with immunochemotherapy involving rituximab plus cyclophosphamide, doxorubicin, vincristine, and prednisolone (R‐CHOP), the remainder succumb to the disease 19. A high international prognostic index, extranodal lesions, a non‐germinal center B‐cell phenotype, *BCL2* expression, the deletion of *CDKN2A*, and *MYC* rearrangements have been recognized as high‐risk features in DLBCL treated using R‐CHOP 20. The prognostic and biological significance of *MYC* rearrangements in DLBCL has been thoroughly investigated. However, the role of *PVT1*, which may be co‐amplified with *MYC*, remains unclear.

We herein report the AMU‐ML2 novel DLBCL cell line, obtained from a primary refractory patient. This cell line is uniquely characterized by a HSR at the 8q24 locus, containing *MYC* and *PVT1* and displaying a high expression of *PVT1* long noncoding RNAs. Here, we report the genetic and biological characteristics of this cell line in comparison with other B‐cell lymphoma cell lines.

## Materials and methods

### Case history

A 64‐year‐old man was referred to the clinic presenting pancytopenia, a 2‐month history of anorexia, and general fatigue in November 2011. The laboratory and chest X‐ray examinations revealed a white blood cell count of 1000 μL^−1^, hemoglobin of 9.6 g·dL^−1^, a platelet count of 5000 μL^−1^, LDH of 2397 U·L^−1^, and bilateral pleural effusion (Fig. S1A,B). Abnormal lymphocytes with Burkitt‐like morphology were observed in the pleural effusion and bone marrow (Fig. S1C,D). The patient was diagnosed with DLBCL and commenced treatment with R‐CHOP. G‐banding of cells revealed a complex karyotype; however, fluorescence *in situ* hybridization (FISH) using a *MYC/IGH* probe set revealed a significant increase in *MYC* copy number, in the absence of a fusion signal. Subsequently, the patient underwent a more intensive regimen involving rituximab plus hyperfractionated cyclophosphamide, doxorubicin, vincristine, and dexamethasone, alternating with high‐dose methotrexate and cytarabine (R‐Hyper‐CVAD/MA) 21 after one cycle of R‐CHOP. The patient responded to the treatment; however, he developed cytomegalovirus pneumonia after four cycles of R‐Hyper‐CVAD/MA and died of *Trichosporon asahii* sepsis at 6 months postdiagnosis.

### Establishment of the AMU‐ML2 cell line

The patient provided written informed consent for his cells from the pleural effusion to be used in a procedure approved by the Institutional Review Board of Aichi Medical University. The study methodologies conformed to the standards set by the Declaration of Helsinki. The cells were collected at the time of initial diagnosis, prior to chemotherapy. The cells were cultured in RPMI 1640 medium (Sigma‐Aldrich, St. Louis, MO, USA) supplemented with 10% heat‐inactivated fetal bovine serum (Thermo Electron, Melbourne, Vic., Australia) and 1% penicillin/streptomycin (GIBCO‐BRL, Grand Island, NY, USA). Cultures were maintained at 37 °C in 5% CO_2_, and the medium was partially exchanged every 5–7 days. After 2 months in culture, cell proliferation became continuous. The cell line was designated as AMU‐ML2 after confirmation that cells had started growing again after the conventional freeze–thaw procedure.

### B‐cell lymphoma cell lines, cell culture, and drugs

The AMU‐ML2, SU‐DHL‐10, Raji, P3HR‐1, VAL, and Farage B‐cell lymphoma cell lines were cultured as previously described 22. Prednisolone, cyclophosphamide, vincristine, and doxorubicin were purchased from Wako Pure Chemical Industries (Osaka, Japan).

### Chromosomal analyses and spectral karyotyping

Chromosome preparations for G‐banding, spectral karyotyping, and FISH were performed according to standard procedures (SRL Inc., Tokyo, Japan) 23, 24. A total of 20 metaphase spreads were analyzed by G‐banding, and the karyotype was defined according to the International System for Human Cytogenetic Nomenclature 25. Spectral karyotyping analyses were performed in metaphase spreads according to standard procedures (SRL Inc.) 26.

### Morphology and immunophenotype of bone marrow‐ and pleural effusion‐derived patient cells

Cells obtained from the patient's bone marrow and a pleural effusion were air‐dried on a glass slide. The cellular morphology was analyzed using May–Grunwald–Giemsa and immunohistochemistry (IHC) staining. The following antigens were examined by flow cytometry (SRL Inc.): CD2, CD3, CD4, CD5, CD7, CD8, CD10, CD19, CD20, CD34, and CD56. The CD3, CD5, CD10, CD20, CD79a, cyclin D1, BCL2, BCL6, TP53, TdT and MIB‐1 labeling indices were examined by IHC. The anti‐TP53 antibody was purchased from DAKO Japan Inc. (clone DO‐7; Kyoto, Japan). The Epstein–Bar virus‐encoded RNA was examined by *in situ* hybridization.

### FISH analysis

Detection of the 8q24 aberration and deletion of 17p in both metaphase and interphase nuclei was performed using the Vysis LSI IGH/MYC/CEP8 Tri‐Color Dual Fusion Probes (Fig. S1A) and the Vysis LSI TP53/CEP17 Dual Fusion Probes (Abbot Molecular, Des Plaines, IL, USA). Bacterial artificial chromosome (BAC) and P1‐derived artificial chromosome (PAC) clones RP1‐160A22, RP1‐193B12, RP1‐109F14, and RP11‐55J15 were obtained from Invitrogen (Carlsbad, CA, USA). FISH probes for the analysis of chromosome 6p and 8q breakpoints were prepared with DNA extracted from BAC clones and Poseidon Sub‐Telomeric Probes for chromosome 8 (KREATECH, Amsterdam, the Netherlands) (Fig. S1A,B). DNA extraction was performed using the NucleoBond Xtra Midi kit (Macherey‐Nagel, Düren, Germany). Labeling and hybridization of DNA were performed as previously described 27. BAC/PAC information was obtained from the National Center for Biotechnology Information website (https://www.ncbi.nlm.nih.gov/genome/gdv/), and the probes were confirmed to map to the precise chromosomal bands using metaphase spreads from peripheral blood lymphocytes of healthy donors.

### Genome copy number analyses

Array‐CGH was performed using the SurePrint G3 Human CGH 2 × 400K, Oligo Microarray Kit (G4448A; Agilent Technologies, Santa Clara, CA, USA), which contains approximately 400 000 60‐mer oligonucleotides covering the entire human genome. Genomic DNA was extracted from AMU‐ML2 cells using the QIAamp DNA Mini Kit (Qiagen, Hilden, Germany). DNA labeling, hybridization, and washing were performed according to the manufacturer's protocols. Scanning analyses were performed using the Agilent Microarray Scanner (Agilent Technologies). The array data were analyzed to determine chromosomal copy numbers using the feature extractions version 11.0 software program (Agilent Technologies) and the analytical software program dna analytics, version 4.0 (Agilent Technologies). The ADM‐1 algorithm (Threshold 6.0) was adopted to detect genomic aberrations 28. The relationship between the log_2_ ratio (X), the copy number in the reference sample (A), the average copy number in tumor cells (B), and the ratio of tumor cells (C) was determined as X = Log_2_.

### DNA sequence analyses

Total RNA was isolated from AMU‐ML2 cells using the NucleoSpin RNA kit (TaKaRa Bio, Inc., Tokyo, Japan). Complementary DNA was synthesized from 2 μg of total RNA by using the High‐Capacity cDNA Reverse Transcription Kit (Invitrogen). PCR amplification of the open reading frame of *TP53* was performed with a gene‐specific primer set as follows: forward primer, 5′‐ATGGAGGAGCCGCAGTCAGA; reverse primer, 5′‐TCAGTCTGAGTCAGGCCCTT. Sequence analysis was performed using the BigDye Terminator v3.1 Cycle Sequencing Kit and an Applied Biosystems 3130 Genetic Analyzer (Foster City, CA, USA).

### Western blot analyses

Western blot analyses were performed as described previously 29. Briefly, proteins in the cell lysate were separated on 10% polyacrylamide gels, followed by transfer onto a polyvinylidene fluoride membrane (Merck Millipore, Darmstadt, Germany). The membrane was hybridized with a mouse monoclonal anti‐c‐Myc antibody (9E10; Wako) and a rabbit anti‐β‐actin antibody (13E5; Cell Signaling Technologies, Danvers, MA, USA). The membrane was visualized using ImmunoStar LD (Wako).

### Real‐time qRT‐PCR

The expression levels of the *MYC* and *PVT1* transcripts were quantified by real‐time qRT‐PCR using TaqMan Gene Expression Assays (*MYC*, Hs00153408_m1; *PVT1*, Hs00413039_m1; β‐actin, Hs99999903_m1; Applied Biosystems) and the StepOnePlus™ Real‐Time PCR System (Applied Biosystems). In brief, total RNA was extracted using the RNeasy Mini Kit (Qiagen) and cDNA was synthesized from total RNA by using the SuperScript III First‐Strand Synthesis System (Invitrogen). The expression level of *circPVT1* was quantified by real‐time qRT‐PCR using SYBR Green I (TaKaRa Bio, Inc.) with the StepOnePlus™Real‐Time PCR System, as previously described 13, 30, 31. cDNA was amplified using KOD FX Neo polymerase (Toyobo, Tokyo, Japan) with SYBR Green I. Primers for *circPVT1* were follows: forward: 5′‐GGTTCCACCAGCGTTATTC; reverse: 5′‐CAACTTCCTTTGGGTCTCC 32. *GAPDH* was used as an internal standard for the SYBR Green I‐based analysis. Relative expression was determined using the 2^−ΔΔCT^ method. The relative gene expression was determined using the gene expression in peripheral blood leukocytes (PBLs) from healthy donors as a control.

### MYC knockdown

The pRetrosuper Myc shRNA and pMKO.1‐puro GFP shRNA were gifts from Addgene (plasmids #15662 and #10675, respectively; Cambridge, MA, USA) 33, 34. To obtain cells stably expressing decreased levels of MYC, the MYC shRNA vector (AMU‐ML2/MYCsh) or the control GFP shRNA vector (AMU‐ML2/GFPsh) was introduced into AMU‐ML2 cells. The retroviral plasmids were packaged into 293T cells using the pCL10A vector. Viral supernatants were harvested 96 h after transfection and filtered before infection. The cells were infected with retroviruses in the presence of 8 μg·mL^−1^ Polybrene (Sigma‐Aldrich). Antibiotic selection (puromycin; 0.3 μg·mL^−1^; Wako) was begun 48 h after infection and continued for at least 3 days. Following infection and antibiotic selection, the cells were examined for MYC protein levels using western blotting.

### Microarray gene expression analyses

The experimental procedure for the cDNA microarray analysis was based on the manufacturer's protocol (Agilent Technologies). In brief, cDNA synthesis and cRNA labeling with the cyanine 3 (Cy3) dye were performed using the Agilent Low Input Quick Amp Labeling Kit. The Cy3‐labeled cRNA was purified, fragmented, and hybridized on a Whole Human Genome 4 × 44k Oligo Microarray Chip containing 43 377 oligonucleotide probes, using a Gene Expression Hybridization kit. The microarray slide was washed and scanned using an Agilent DNA microarray scanner (Agilent Technologies). The scanned data were quantified using the feature extraction software program, version 11.0.1.1 (Agilent Technologies). The signal intensities were then normalized as described elsewhere 35.

The background signals were also normalized, and the microarray expression data were rank‐ordered based on the differential expression in AMU‐ML2/MYCsh cells versus AMU‐ML2/GFPsh cells as follows: Up‐ and downregulated genes were called when exhibiting a > 0.27 increase (fold change > 2.0) or < −0.27 decrease (fold change < 0.5), respectively, in AMU‐ML2/MYCsh cells versus AMU‐ML2/GFPsh cells.

## Results

### Karyotyping and FISH analysis of 8q24 abnormalities in AMU‐ML2 cells

**Figure 1:**
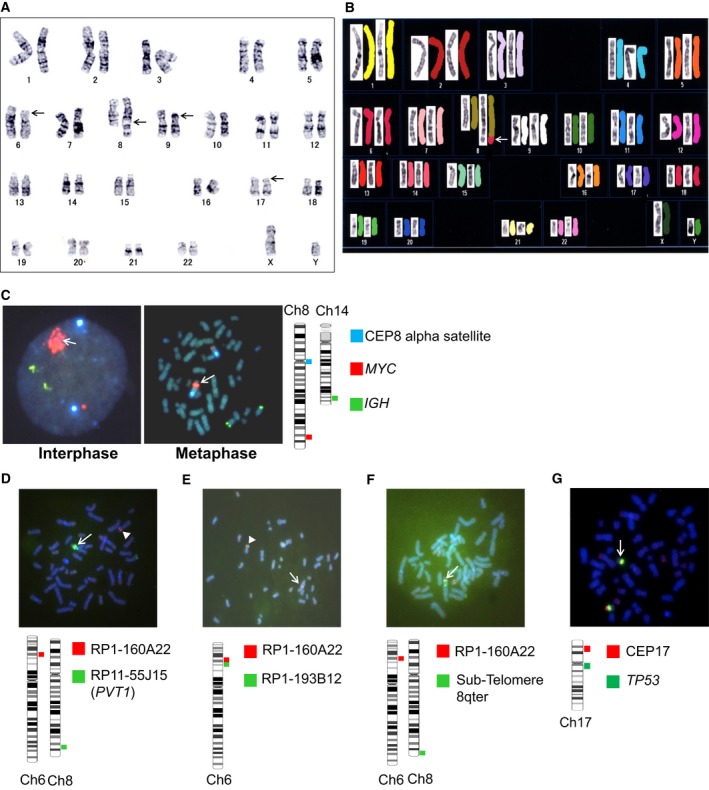
Karyotype and FISH analyses of AMU‐ML2 cells. (A, B) Representative karyotype by G‐banding and spectral karyotyping indicated 46,XY,del(6)(p21p23),der(8)(8pter→8q24::hsr::6p21→6pter),add(9)(p13),del(17)(p?). (A) The black arrows indicate abnormal sites detected by G‐banding. (B) Spectral karyotyping analysis; the white arrow indicates the fusion of part of chromosome 6 (6ptel–6p21, red) with the telomeric region of 8q24. (C–F) FISH analysis of 8q24 containing the entire *MYC* and *PVT1* regions (C), the 6p22–p21 breakpoint of t(6;8) (D–F) and the 17p13.2 locus, containing the *TP53* gene (G) in AMU‐ML2 cells. Schematic illustrations of the FISH probes used in this study are presented beside each figure panel. (C) Interphase (left) and metaphase (right) FISH analyses using Vysis LSI IGH (green), MYC (red), and CEP8 (aqua) Tri‐Color Dual Fusion Probes are shown. The white arrow indicates the HSR on the MYC probe (red), without any *IGH*/*MYC* fusion signals. The copy number of the amplicon at 8q24.21 was approximately 26 per tumor cell. (D) The white arrow indicates the HSR on RP11‐55J15 (green), which covers part of *PVT1* but not *MYC*, as depicted in Fig. S2A. The white arrowhead indicates a single copy signal on RP1‐160A22 (red) at 6p22. (E) The white arrowhead indicates a fusion signal on the normal chromosome 6. RP1‐160A22, shown in red; RP1‐193B12, shown in green. The white arrow indicates another red signal but no green signal on the derivative chromosome 8. (F) The white arrow indicates a fusion signal on the derivative chromosome 8. RP1‐160A22, shown in red; KREATECH Sub‐Telomere 8qter, shown in green. (G) The white arrow indicates a single copy signal at the *TP53* gene locus. Vysis LSI CEP17, show in green; *TP53* probe, shown in red. Ch, chromosome.

The karyotyping of AMU‐ML2 cells by G‐banding and spectral karyotyping revealed the following karyotype: 46,XY,del(6)(p21p23),der(8)(8pter→8q24::hsr::6p21→6pter),add(9)(p13),del(17)(p?) 21 (Fig. 1A,B). The FISH analysis of AMU‐ML2 cells using a *MYC/IGH* probe set revealed no fusion signal for *IGH* and *MYC*; however, a significant increase in the *MYC* copy number was observed, corresponding to the HSR on chromosome 8q24 (Fig. 1C). The copy number of the amplicon at 8q24.21 was approximately 26 per tumor cell. A FISH analysis using RP11‐55J15, containing a segment of *PVT1* but not the *MYC* gene (Fig.  S2A), also showed a significant increase in the copy number at the *PVT1* gene locus (Fig. 1D). The FISH analysis of AMU‐ML2 cells using an RP1‐160A22*/*RP11‐55J15 probe set revealed that chromosome 6p21.2‐22.2 translocated on chromosome 8q (white arrow), and a significant increase in the *PVT1* copy number was observed (white triangle), corresponding to the HSR on chromosome 8q24 (Fig. 1D). RP11‐55J15 contained a segment of *PVT1* but not the *MYC* gene (Fig. S2A), and also showed a significant increase in the copy number at the *PVT1* gene locus. The FISH analysis of AMU‐ML2 cells using a RP1‐160A22*/*RP1‐193B12 probe set revealed fusion signal for both probes on chromosome 6p (white triangle); however, the other RP1‐193B12 was absent on chromosome 6p, and the other RP1‐160A22 translocated on chromosome 8q (white arrow) (Fig. 1E). The FISH analysis of AMU‐ML2 cells using a RP1‐160A22*/*Sub‐Telomere 8qter probe set revealed a fusion signal for both probes on chromosome 8q telomere (Fig. 1F). The FISH analysis of AMU‐ML2 cells using a TP53*/* CEP17 set revealed that one of TP53 disappeared on chromosome 17p (Fig. 1G). The copy number of the amplicon at 8q24.21 was approximately 26 per tumor cell.

### Array‐CGH analyses and *TP53* sequencing of AMU‐ML2 cells

**Figure 2:**
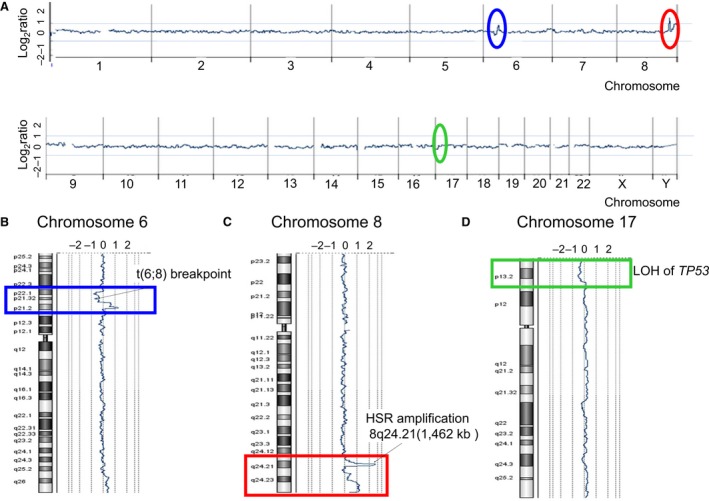
High‐resolution aCGH analysis in AMU‐ML2 cells. Genomewide copy number aberrations in AMU‐ML2 cells were determined using an Agilent SurePrint G3 Human CGH 2× 400K Oligo Microarray (Agilent Technologies). The median probe spacing was approximately 4.6 kb. (A) Summary of the aCGH analysis. The *x*‐axis indicates the chromosome number, whereas the *y*‐axis indicates the log_2_ ratio (copy number aberrations). The red oval indicates a 1462‐kb highly amplified region, containing *MYC* and *PVT1* at 8q24.21. The blue and green ovals indicate copy number changes at 6p22 to 6p21 and at 17p13, respectively. The other copy number alterations (CNAs) detected are summarized in Table 1. (B) The aCGH analysis shows a 7431‐kb deletion at 6p22.1–6p21.31, where the t(6;8) breakpoint was detected by FISH, as indicated in Fig. 4F. Blue rectangle, copy number changes at 6p22–6p21. (C) A 1462‐kb amplification detected by the aCGH analysis, containing *MYC* and *PVT1* at 8q24.21, where 8q24.1 HSR was detected by FISH, as indicated in Fig. 4C. Red rectangle, copy number changes at 8q24. (D) A 7522‐kb deletion at 17p13.3–17p.13.1 detected by the aCGH analysis, where a single copy deletion of *TP53* was detected by FISH, as indicated in Fig. 4F. Green rectangle, copy number changes at 17p13.

To further investigate the genomewide copy number aberrations in AMU‐ML2 cells, we performed an array‐CGH (aCGH) analysis using an Agilent human CGH 400K Oligo Microarray format (Agilent Technologies; Fig. 2A, Table 1). The aCGH analysis identified several large deletions and amplifications as follows: a 7431‐kb deletion at 6p22.1–6p21.31, which contains the t(6;8) breakpoint detected by FISH (Fig. 2B); a 1462‐kb amplification at 8q24.21, which contains *MYC* and *PVT1* (Fig. 2C); and a 7522‐kb deletion at 17p13.3–17p.13.1, which harbors the *TP53* gene (Fig. 2D). The aCGH analysis also showed that the *HIST1* gene locus, spanning 27.9–35.3 Mb on 6p22–6p21, contained a monoallelic deletion (Fig. 2B). Furthermore, our aCGH analysis detected 14 additional copy number alterations (CNAs). Segment gains were detected on chromosomes 6p21.31–p21.2, 8p11.23, 8q24.21, 8q24.3, 9p24.3–9p13.1, 14q11.2, and 19q13.2, whereas segment losses were detected on chromosomes 6p25.3, 6p22.1–6p21.31, 6p12.3, 7q31.33, 14q32.33, and 17p13.3–17p.13.1 (Table 1).

**Table 1:** Copy number alteration (CNA) regions and estimated target genes detected by array‐CGH in AMU‐ML2 cells

Chromosome band	Start (kb)	End (kb)	Size (kb)	Average log_2_ ratio	Number of genes	Candidate genes^a^
Gains						
6p21.31–6p21.2	35 339	38 624	3285	0.62	39	*MAPK14, MAPK13, ETV7, PIM1*
	38 629	39 800	1171	1.21	9	
8p11.23	39 354	39 505	151	1.07	0	
8q24.21	126 515	128 586	2071	0.55	2	
8q24.21	128 611	130 073	1462	2.62	2	*MYC, PVT1*
8q24.3	138 653	146 147	7494	1.04	82	*EIF2C2, BOP1, MAPK15, MAF1*
9p24.3–9p13.1	189	390 481	390 292	0.45	128	*JAK2, PAX5*
14q11.2	21 523	22 237	714	0.57	2	
19q13.2	46 947	47 693	746	0.54	22	*LYPD4, CD79A*
Losses						
6p25.3	158	318	160	−0.54	1	*DUSP22*
6p22.1–6p21.31	27 896	35 327	7431	−0.52	133	*HIST1H2AK, HIST1H2AL, HIST1H3I, HIST1H4L, HIST1H3J, HIST1H2AM, HIST1H2BO, HLA‐E, NOTCH4, HLA‐A, HLA‐B, HLA‐C, HLA‐DRA, HLA‐DRB5, HLA‐DRB1, HLA‐DQA1, HLA‐DQB1, HLA‐DQA2,*
6p12.3	50 840	50 944	104	−0.7	2	*TFAP2D, TFAP2B*
7q31.33	124 941	125 414	473	−0.83	0	
14q32.33	105 314	106 037	723	−1.12	0	
17p13.3–17p13.1	51	7522	7471	−0.45	110	*TP53*

a Genes listed here are candidates based on their putative function.

In addition, reverse transcription‐polymerase chain reaction (RT‐PCR) and Sanger sequencing analysis detected a frameshift mutation of *TP53* in AMU‐ML2 cells (c.377_378delAC; Fig. S5).

### *MYC, PVT1,* and *circPVT1* mRNA levels in AMU‐ML2 and other B‐cell lymphoma cell lines

**Figure 3:**
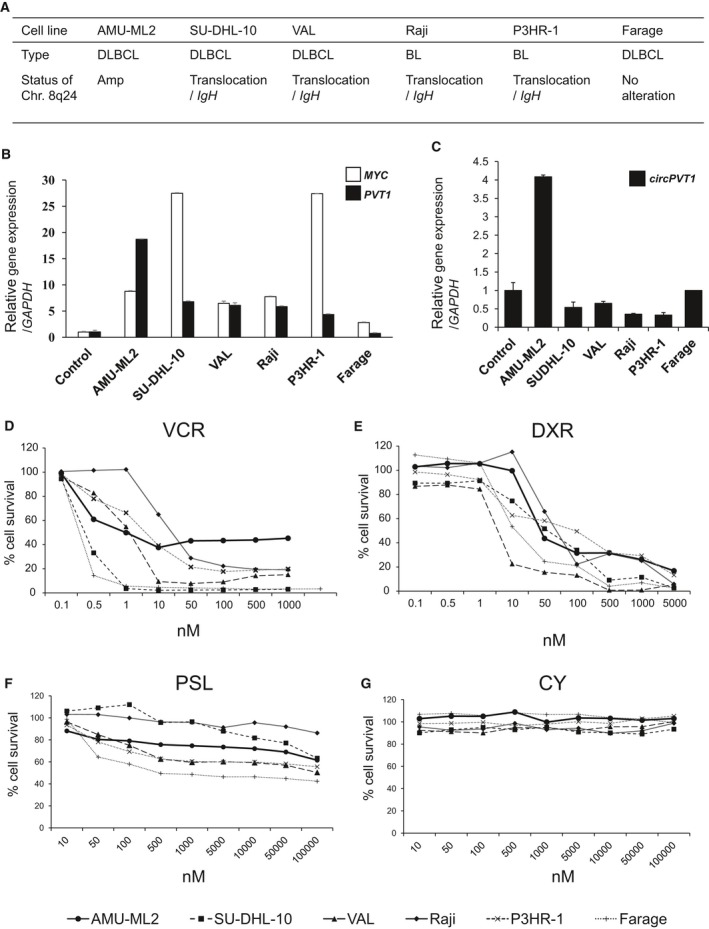
*MYC* and *PVT1* expression and the effect of chemotherapy on cell survival in AMU‐ML2 and other B‐cell lymphoma cell lines. (A) Chromosome 8q24 status in the B‐cell lymphoma cell lines used in this study. Amp, amplification. (B) *MYC* and *PVT1* expression in B‐cell lymphoma cell lines (AMU‐ML2, SU‐DHL‐10, VAL, Raji, P3HR‐1, and Farage) and normal PBLs using TaqMan probe methodology. No association between the relative mRNA expression of *MYC* and that of *PVT1* is observed. The relative gene expression is shown after normalization to *GAPDH*. The data are expressed relative to the mRNA levels found in the corresponding PBL samples, arbitrarily defined as 1. The values shown represent the means ± SE (*n* = 3). (C) *circPVT1* expression in B‐cell lymphoma cell lines as determined by real‐time qRT‐PCR using SYBR Green methodology. The expression of *circPVT1* in AMU‐ML2 cells was the highest among the cell lines used in this study. (D–G) Effect of chemotherapy on cell survival in AMU‐ML2 and other B‐cell lymphoma cell lines. AMU‐ML2 and other B‐cell lymphoma cell lines (SU‐DHL‐10, VAL, Raji, P3HR‐1, and Farage) were treated with the indicated concentration of vincristine (VCR, C) (1000, 500, 100, 50, 10, 1, 0.5, or 0.1 nm), doxorubicin (DXR, D) (5000, 1000, 500, 100, 50, 10, 1, 0.5, or 0.1 nm), prednisolone (PSL, E) (100 000, 50 000, 10 000, 5000, 1000, 500, 100, 50, or 10 nm), and cyclophosphamide (CY, F) (the same as prednisolone) for 72 h. After incubation, the cells were assayed using the MTT assay. Data are expressed relative to the mean optical density (595 nm) found in untreated cells, which was arbitrarily defined as 100%. Data are expressed as the means ± SE (*n *= 3).

To investigate the influence of the 8q24.21 amplification on *MYC*,* PVT1,* and *circPVT1* expression, we performed quantitative RT‐PCR (qRT‐PCR) analysis in AMU‐ML2 cells and other B‐cell lymphoma cell lines, for which the chromosomal status at 8q24 is summarized in Fig. 3A. qRT‐PCR analysis showed that the expression of *MYC, PVT1,* and *circPVT1* was significantly higher in AMU‐ML2 cells than that in PBLs from healthy donors. In addition, the expression of *PVT1* and *circPVT1* in AMU‐ML2 cells was the highest among the cell lines used in this study (Fig. 3B,C).

### Resistance of AMU‐ML2 cells to vincristine

To clarify the effect of anticancer drugs used in the chemotherapy of DLBCL on AMU‐ML2 cells, we performed an MTT assay using AMU‐ML2 and other B‐cell lymphoma cell lines following treatment with vincristine, doxorubicin, prednisolone, and cyclophosphamide. The MTT assay showed that AMU‐ML2 cells exhibited resistance to vincristine (at 100, 500, and 1000 nm), whereas cell survival in other B‐cell lymphoma cell lines was almost completely suppressed in a dose‐dependent manner (Fig. 3D). The MTT assay also showed that doxorubicin dose‐dependently decreased the cell survival rate in all cell lines used in this study (Fig. 3E). Prednisolone partially suppressed cell proliferation in AMU‐ML2 and B‐cell lymphoma cell lines, whereas cyclophosphamide did not exhibit any tumor‐suppressive effects (Fig. 3F,G).

### The role of MYC in proliferation and gene expression in AMU‐ML2 cells

**Figure 4:**
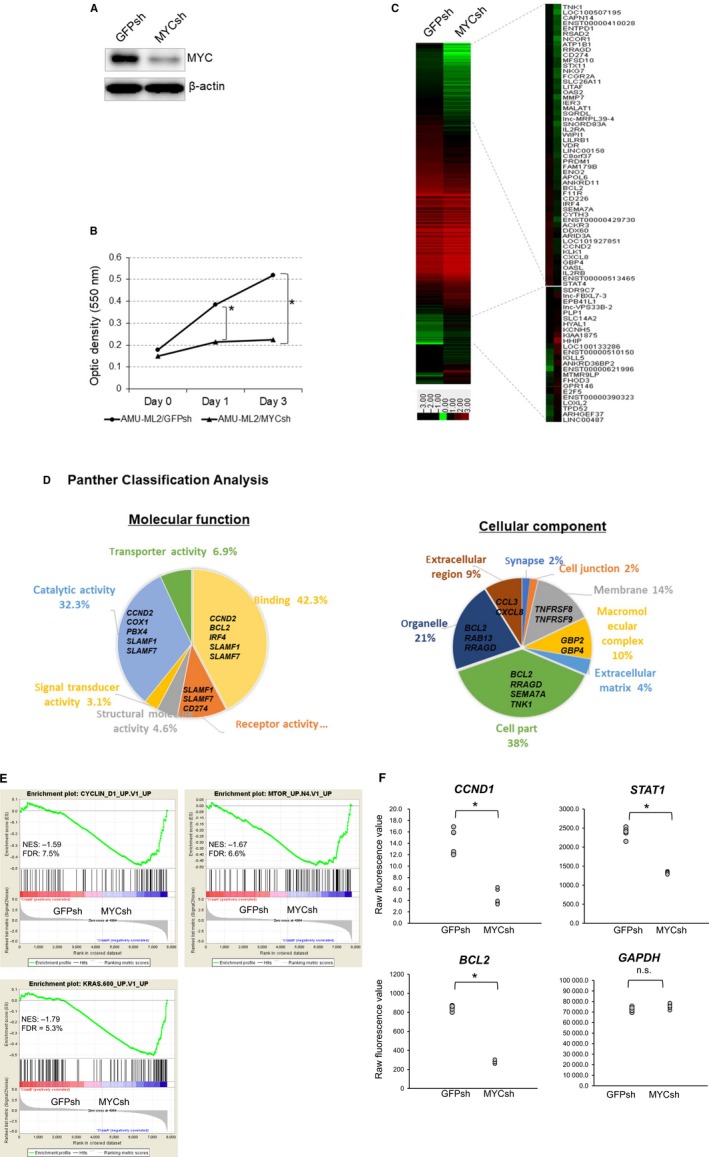
Effect of MYC knockdown on cell proliferation and gene expression in AMU‐ML2 cells. (A) The *MYC* gene silencing shRNA vector (pRetrosuper Myc shRNA, plasmid 15662 from Addgene) or control GFPsh vector (pMKO.1‐puro GFP shRNA, plasmid 10675 from Addgene) was stably introduced into AMU‐ML2 cells using retroviral transduction. Cell clones were obtained after puromycin selection and subsequent single‐cell cloning. Five micrograms of cell lysate was subjected to a western blot analysis, to detect the MYC protein. β‐Actin was used as an internal control. (B) MTT analysis of the growth rate of AMU‐ML2/GFPsh and AMU‐ML2/MYCsh clones. The optical density (595 nm) at each time point (days 0, 1, and 3) is presented as the means ± SE (*n *= 4). Statistical significance between groups was determined using Student's *t*‐test. Statistical analyses were performed using spss 23.0 program (SPSS Inc.). The asterisk (*) indicates significant differences at *P* < 0.05, compared to the GFPsh clone. (C–E) Gene expression analysis. Total RNA from MYCsh and GFPsh clones was extracted and subjected to a cDNA microarray analysis using a SurePrint G3 Human 8 × 60K V3 format (Agilent Technologies). (C) Heatmap of downregulated (172 genes, fold change < 0.5) and upregulated genes (214 genes; fold change, > 2.0) following MYC knockdown. The heatmap was constructed using normalized values for each sample and the treeview software 43. The corresponding gene names are annotated on the right. (D) Gene ontology analyses using the PANTHER classification system. The downregulated genes were classified using the PANTHER‐Gene List Analysis (http://www.pantherdb.org). Pie chart showing the percentages of genes classified into each molecular pathway and/or cellular component. (E) The GSEA was conducted using the gsea software program, v2.2.4, and Molecular Signatures Database (Broad Institute). All raw data were formatted and applied to oncogenic signatures (C6). Representative GSEA enrichment plots and corresponding heatmap images of the indicated gene sets are shown for the MYCsh and GFPsh clones, respectively. Genes contributing to the enrichment are shown in rows, and the samples are shown in columns on the heatmap. Expression is shown as a gradient from high (red) to low (blue). FDR, false discovery rate; NES, normalized enrichment score. (F) Graphs showing the differential gene expression in cells expressing GFPsh and MYCsh. Raw fluorescence values obtained by scanning were utilized for the comparison of gene expression. The gene oligonucleotide probes corresponding to cyclin D1, *STAT1,* and *BCL2* are shown. *P < 0.05, significant difference.

As our data showed that *MYC* expression was higher in AMU‐ML2 cells than that in PBLs from normal healthy volunteers, we next investigated the effects of MYC expression on cell proliferation using RNA interference. Our western blot analysis showed robust MYC expression in AMU‐ML2 cells expressing the control GFPsh vector; the expression of this protein decreased in cells expressing MYCsh (Fig. 4A). Therefore, we examined the effect of MYC knockdown on cell proliferation using an MTT assay. The MTT assay showed that the optical densities reflecting the cell numbers were significantly higher at both days 1 and 3 in cells expressing GFPsh than in those expressing MYCsh, strongly suggesting that *MYC* expression is closely associated with cell proliferation in AMU‐ML2 cells (Fig. 4B).

To further investigate the role of MYC in tumorigenesis in AMU‐ML2 cells, we performed a comprehensive gene expression analysis using Agilent cDNA microarrays. A heatmap analysis revealed different gene expression patterns between cells expressing GFPsh and those expressing MYCsh (Fig. 4C). We also observed that *MYC* knockdown downregulated the expression of 172 genes by < 0.5‐fold and upregulated the expression of 214 genes by > 2.0‐fold compared with that in cells expressing GFPsh (Tables S1 and S2). Moreover, we found that *MYC* knockdown significantly decreased the expression of cyclin D1 (*CCND1*), *BCL2*, and *STAT1* but not that of *GAPDH* in AMU‐ML2 cells (Fig. 4F). By using a PANTHER classification analysis, we showed that 42.3% of the downregulated genes encoded binding proteins, including *CCND2*,* BCL2*,* IRF4*,* SLAMF1,* and *SLAMF7* (Fig. 4D,E). Notably, this analysis also showed that 8.0% of the downregulated genes encoded either inflammation‐ or apoptosis‐related signaling molecules (Fig. S3). Therefore, we performed a gene set enrichment analysis (GSEA) to investigate whether the expression of a specific set of oncogenesis‐associated genes significantly differed between the MYC knockdown and control cells. Genes with oncogenic signatures showed a significant inactivation of cyclin D signaling‐related genes (*NKG7*,* IFITM1*, and *PDE2*), of genes induced by mTOR signaling (*ITPR1*,* ATF3,* and *BATF*), and of Ras signaling‐associated genes (*NR4A3*,* DOCK4,* and *SATB1*) (Fig. 4E). Furthermore, a GSEA using the Kyoto Encyclopedia of Genes and Genomes database showed a significant inactivation of genes associated with peroxisome proliferator‐activated receptor‐, hematopoietic‐, and cytokine–cytokine receptor‐related signaling (Fig. S4). Collectively, these results suggest that *MYC* expression is closely associated with tumor cell growth in AMU‐ML2 cells.

## Discussion

A HSR is occasionally observed in solid tumors, but rarely detected in DLBCL 36. In this study, we established a novel DLBCL cell line, AMU‐ML2, using patient cells, which was uniquely characterized by a HSR at the 8q24 locus, containing *MYC* and *PVT1* (Fig. 1). Our aCGH analysis clearly identified the 8q24 amplicon size as ranging from 128 611 to 130 073 kb in the HSR (Fig. 2, Table 1). Moreover, we found that the amplicon contained the entire *MYC* and *PVT1* sequences and was amplified at more than 20 copies per cell. To our knowledge, this is the first to report of a DLBCL cell line showing an amplicon containing the entire *MYC* and *PVT1* genes at 8q24. As the patient‐derived cells were collected before the initiation of chemotherapy and established within 2 weeks, the detected chromosomal aberrations reflect the actual pathogenesis for the onset of aggressive DLBCL and do not reflect chemotherapy and/or long‐term cell culture.

The expression levels of both *MYC* and *PVT1* were significantly higher in AMU‐ML2 and other B‐lymphoma cells with 8q24 abnormalities than those in PBLs from healthy donors and Farage cells lacking 8q24 abnormalities (Fig. 3B). The expression of *MYC* and *PVT1* appears similar to that observed in gene amplification and translocation events at immunoglobulin loci.

*MYC*, a candidate oncogene at the 8q24 amplification, has been reported to play an important role in the pathogenesis of lymphoma and leukemia 37. In this study, we show that the *MYC‐*shRNA‐mediated knockdown significantly suppressed cell proliferation in AMU‐ML2 cells (Fig. 4A,B). Furthermore, a cDNA microarray analysis revealed that the *CCND2* cell‐cycle‐promoting gene, the *IRF4* oncogenic transcription factor, and the *BCL2* anti‐apoptotic gene were all downregulated following *MYC* knockdown (Fig. 4C). Although MYC is assumed to repress the transcription of *BCL2* directly or through p53, *BCL2* expression was repressed by MYC inhibition in AMU‐ML2 cells. This suggests the dysfunction of p53 in the AMU‐ML2 background. Our GSEA also showed that *MYC* knockdown inactivated gene expression for oncogenic gene sets, including the Ras, mTOR, and cyclin D signaling pathways (Fig. 4E). These results strongly suggest that the survival and proliferation of AMU‐ML2 cells strongly depend on the aberrant *MYC* expression.

Recent reports have suggested that the deregulation of *PVT1* consequent to gene amplification and chromosomal translocation may contribute to tumorigenesis and drug resistance 9, 38. Patients with DLBCL often suffer from resistance to chemotherapy, including R‐CHOP. Resistance to cisplatin by *PVT1* overexpression has been reported in gastric and ovarian cancers 39. In addition, it has been reported that *PVT1* promotes the development of multidrug resistance by mediating the mTOR/HIF‐1α/P‐glycoprotein pathway and/or the MRP1 signaling pathway 40. Moreover, overexpression of *circPVT1* has been recently shown to serve as a prognostic factor in gastric cancer 14. In the present study, we found that AMU‐ML2 cells displayed vincristine resistance, in contrast to other B‐cell lymphoma cell lines (Fig. 3D). As the expression of *PVT1* and *circPVT1* in AMU‐ML2 cells was the highest among the cell lines tested (Fig. 3B,C), the overexpression of *PVT1* and *circPVT1* may contribute to vincristine resistance in AMU‐ML2 cells. Although the pathogenic role of *PVT1* in lymphoma is not precisely known, the examination of whether the microRNAs and/or *circPVT1* transcribed from the *PVT1* locus are linked to tumorigenesis and drug sensitivity in AMU‐ML2 cells is worthy of further study.

The co‐amplification of *MYC* and *PVT1* has been reportedly observed in several types of solid tumors and is associated with a shortened survival 9. However, the biological differences underlying the deregulation of *MYC* alone, or that of both *MYC* and *PVT1*, are not well characterized in DLBCL. Future clinical studies utilizing RNA‐seq, whole‐genome sequencing, and functional experiments will help clarify the precise incidence, clinical implications, and biological significance of the co‐amplification of *MYC* and *PVT1* in DLBCL.

In addition to the HSR at 8q24, we have identified several other CNA loci (Table 1, Fig. 2A). *TP53* is a well‐known tumor suppressor gene at the 17p13.1 locus. Loss of heterozygosity and inactivating mutations in the *TP53* gene are frequently observed in many cancers and constitute a poor prognosis for DLBCL. In the present study, our aCGH analysis showed a monoallelic 7.8‐Mb deletion, which contains the *TP53* gene (Fig. 2D). Moreover, the identification of a *TP53* frameshift mutation in AMU‐ML2 cells suggested that p53 does not function in these cells. It has been reported that the disruption of the p14/ARF‐MDM2‐p53 pathway that accompanies MYC overexpression contributes to the pathogenesis of BL 41. It may therefore be possible that the pathophysiology of DLBCL is partly associated with the HSR at 8q24 as well as with the dysregulation of p53 in AMU‐ML2 cells. We also detected a chromosomal breakpoint and a single copy deletion in the *HIST1* gene in AMU‐ML2 cells (Table 1, Fig. 2B). The *HIST1* gene spans over 2 Mb and contains all the replication‐dependent H1 histone genes and other core histone genes at the 6p22–p21 locus. Moreover, it encodes histone proteins, which associate with the double‐stranded helical DNA molecule to form the chromatin, and play a role in gene regulation 42. In AMU‐ML2 cells, the deletion of a part of *HIST1*, after the t(6;8) translocation, may result in the haploinsufficiency of *HIST1*, which may influence chromatin remodeling and cellular gene expression.

In conclusion, the present study is the first to show a HSR containing both *MYC* and *PVT1* at the 8q24 locus, in the AMU‐ML2 novel DLBCL cell line. We also demonstrated a loss of heterozygosity for *TP53* at 17p13 with a frameshift mutation of *TP53*, suggesting that the high expression of *MYC* and *TP53* dysfunction may contribute to cell survival in DLBCL. The AMU‐ML2 cell line is useful for investigating the roles of *MYC* and *PVT1* and the interaction of the products of both genes in lymphomagenesis. Furthermore, it would be of interest to investigate the molecular mechanism through which AMU‐ML2 cells induce vincristine resistance. Our finding that *MYC* expression is closely related to the expression of oncogenic genes, including those in the Ras, mTOR, and CCND signaling pathways, provides new insights that may aid in the development of novel molecular‐targeted drugs for the treatment of patients with DLBCL. Further studies are required to clarify the role of *PVT1* in the pathophysiology of DLBCL.

## Author contributions

IH designed the study. SM, AO, SK, JK, and MT performed the experimental analyses. SM, IH, and AO wrote the manuscript. AN, ST, KU, TH, MG, SM, MG, HY, MW, and MS contributed reagents/materials/analysis tools. YH, HM, RU, MN, and AT contributed to overall project management.

## Conflict of interest

The authors declare no conflict of interest.

## Supporting information


**Fig. S1.** Chest X‐ray and computed tomography (CT) findings of the patient with DLBCL on admission. (A) Chest X‐ray and B) CT findings revealing a bilateral moderate to severe pleural effusion. Morphology and immunohistochemistry (IHC) for the pleural effusion and bone marrow (BM) samples in the patient with DLBCL. (C, D) Cells from the pleural effusion and bone marrow showing medium to large cells with Burkitt‐like morphology (E‐H) IHC analysis of the patient‐derived BM cells. BM‐derived cells were incubated with anti‐BCL6 (E), anti‐cyclin D1 (F), anti‐MUM (G), and anti‐BCL2 (H) antibodies according to the manufacturer's instructions. Original magnification: 400×; MG: May–Grunwald–Giemsa staining.

**Fig. S2.** Schema of the detected genomic aberrations and the BAC/PAC probes in the corresponding chromosomal gene locus. (A) Genomic features at 8q24. The amplicon detected by aCGH analysis (green), the gene structure of *MYC* and *PVT1*, the *PVT1*‐encoded microRNAs, BAC clone (RP11‐55J15, green bar), and Vysis FISH probe (LSI/MYC, shown in red) are depicted. The FISH probe for *MYC* (red bar) covers an 821‐kb region containing the entire *MYC* and *PVT1* genes. The RP11‐55J15 BAC clone partially covers the *PVT1* region, but not the *MYC* region. The size of the 8q24 amplicon (green bar) detected by aCGH approximately spans 1462 kb, containing the entire *MYC* and *PVT1* genes. *PVT1* encodes at least six microRNAs (miR‐1204, miR‐1205, miR‐1206, miR‐1207‐5p, miR‐1207‐3p, and miR‐1208; blue bar). The black horizontal bars indicate exons in each gene. (B) Genomic features at 6p22‐p21. The deletion detected by aCGH (purple), gene structure including a *HIST1* gene cluster, and PAC clones (RP1‐97D16, black bar; RP1‐160A22, red bar; RP1‐193B12, green bar; RP3‐408B20 and RP1‐109F14, black bar) used for FISH analysis are depicted. The positional data for genes, microRNAs, and PAC/BAC clones were obtained from the NCBI website (https://www.ncbi.nlm.nih.gov/) and the dna analytics software (Agilent Technologies). The positions (Mb) indicate the distance from the telomeric end on the short arm of each chromosome. Mb, mega base.

**Fig. S3.** Results of Panther Classification Analysis. Gene ontology analyses using the Panther Classification System. The downregulated genes in cells expressing MYCsh were classified using PANTHER‐Gene List Analysis (http://www.pantherdb.org). The percentages of genes classified into each pathway are shown as a pie chart.

**Fig. S4.** GSEA with Kyoto Encyclopedia of Genes and Genomes (KEGG) gene sets. GSEA was conducted using GSEA v2.2.4 software and the Molecular Signatures Database (Broad Institute). All of the raw data were formatted and applied to the KEGG gene sets (C2).

**Fig. S5.** Sequencing analysis of *TP53* gene in AMU‐ML2 cells. (A) Total RNA was isolated from AMU‐ML2 cells using the NucleoSpin RNA kit (TaKaRa Bio, Inc.). After synthesizing complementary DNA, PCR amplification of *TP53* gene was performed with a gene‐specific primer set, as described in Online Supplementary Data. Sequence analysis was performed by using an Applied Biosystems 3130 Genetic Analyzer. The *TP53* frameshift mutation c.377_378delAC was detected in AMU‐ML2 cells (arrowhead). (B) Sequence alignment of *TP53* with wild‐type (WT) *TP53* gene. Nucleotide number is in reference to GenBank accession NM_000546.5 (*TP53* transcript variant 1, mRNA).

**Table S1.** Downregulated genes under *MYC* knockdown in AMU‐ML2 cells.
**Table  S2.** Upregulated genes under *MYC* knockdown in AMU‐ML2 cells.
