# Supplementary material for: Establishment and characterization of a novel vincristine‐resistant diffuse large B‐cell lymphoma cell line containing the 8q24 homogeneously staining region
Source: FEBS Open Bio. 2018 Nov 20;8(12):1977–91. doi: 10.1002/2211-5463.12538 (PMC6275272; doi:10.1002/2211-5463.12538)
Supplement: Supplementary file 1 — Fig. S1. Chest X‐ray and computed tomography (CT) findings of the patient with DLBCL on admission. (A) Chest X‐ray and B) CT findings revealing a bilateral moderate to severe pleural effusion. Morphology and immunohistochemistry (IHC) for the pleural effusion and bone marrow (BM) samples in the patient with DLBCL. (C, D) Cells from the pleural effusion and bone marrow showing medium to large cells with Burkitt‐like morphology (E‐H) IHC analysis of the patient‐derived BM cells. BM‐derived cells were incubated with anti‐BCL6 (E), anti‐cyclin D1 (F), anti‐MUM (G), and anti‐BCL2 (H) antibodies according to the manufacturer's instructions. Original magnification: 400×; MG: May–Grunwald–Giemsa staining. [file FEB4-8-1977-s001.pptx]

## Slide 1
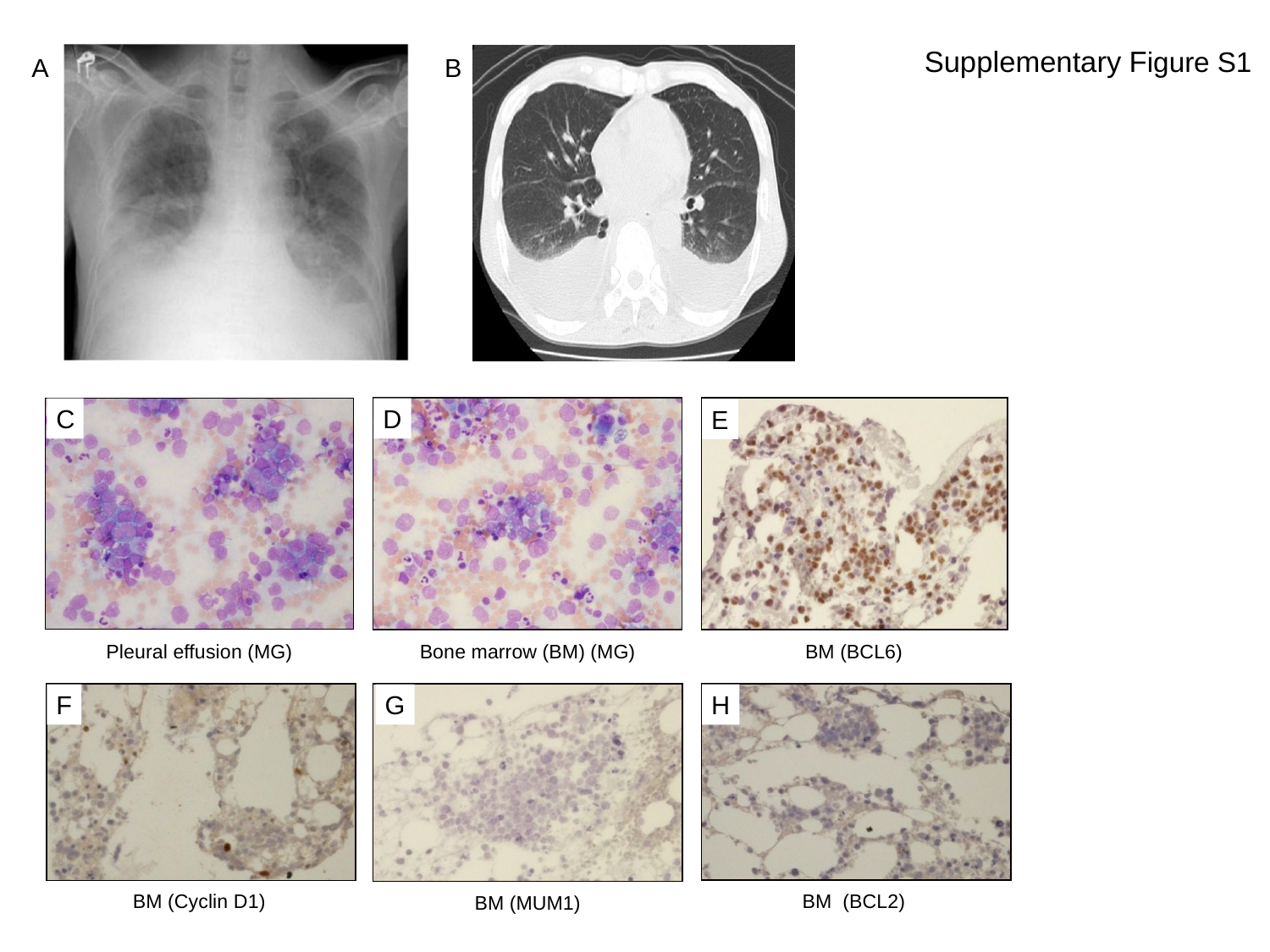

Supplementary Figure S1
A
B
C
Pleural effusion (MG)
D
Bone marrow (BM) (MG)
E
BM (BCL6)
H
BM (BCL2)
G
BM (MUM1)
F
BM (Cyclin D1)

## Slide 2
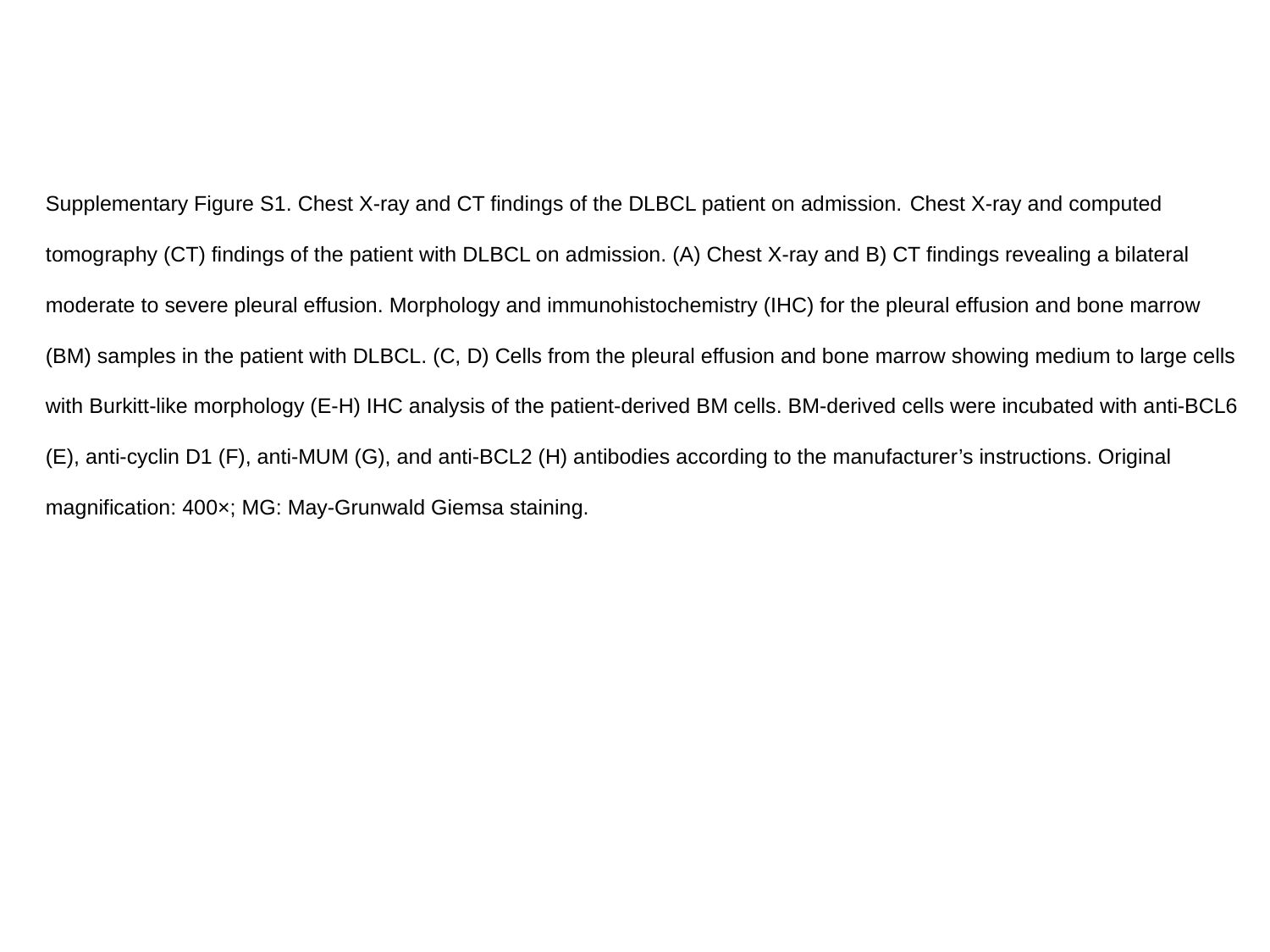

Supplementary Figure S1. Chest X-ray and CT findings of the DLBCL patient on admission. Chest X-ray and computed tomography (CT) findings of the patient with DLBCL on admission. (A) Chest X-ray and B) CT findings revealing a bilateral moderate to severe pleural effusion. Morphology and immunohistochemistry (IHC) for the pleural effusion and bone marrow (BM) samples in the patient with DLBCL. (C, D) Cells from the pleural effusion and bone marrow showing medium to large cells with Burkitt-like morphology (E-H) IHC analysis of the patient-derived BM cells. BM-derived cells were incubated with anti-BCL6 (E), anti-cyclin D1 (F), anti-MUM (G), and anti-BCL2 (H) antibodies according to the manufacturer’s instructions. Original magnification: 400×; MG: May-Grunwald Giemsa staining.
