# Supplementary material for: Establishment and characterization of a novel vincristine‐resistant diffuse large B‐cell lymphoma cell line containing the 8q24 homogeneously staining region
Source: FEBS Open Bio. 2018 Nov 20;8(12):1977–91. doi: 10.1002/2211-5463.12538 (PMC6275272; doi:10.1002/2211-5463.12538)
Supplement: Supplementary file 2 — Fig. S2. Schema of the detected genomic aberrations and the BAC/PAC probes in the corresponding chromosomal gene locus. (A) Genomic features at 8q24. The amplicon detected by aCGH analysis (green), the gene structure of MYC and PVT1, the PVT1‐encoded microRNAs, BAC clone (RP11‐55J15, green bar), and Vysis FISH probe (LSI/MYC, shown in red) are depicted. The FISH probe for MYC (red bar) covers an 821‐kb region containing the entire MYC and PVT1 genes. The RP11‐55J15 BAC clone partially covers the PVT1 region, but not the MYC region. The size of the 8q24 amplicon (green bar) detected by aCGH approximately spans 1462 kb, containing the entire MYC and PVT1 genes. PVT1 encodes at least six microRNAs (miR‐1204, miR‐1205, miR‐1206, miR‐1207‐5p, miR‐1207‐3p, and miR‐1208; blue bar). The black horizontal bars indicate exons in each gene. (B) Genomic features at 6p22‐p21. The deletion detected by aCGH (purple), gene structure including a HIST1 gene cluster, and PAC clones (RP1‐97D16, black bar; RP1‐160A22, red bar; RP1‐193B12, green bar; RP3‐408B20 and RP1‐109F14, black bar) used for FISH analysis are depicted. The positional data for genes, microRNAs, and PAC/BAC clones were obtained from the NCBI website (https://www.ncbi.nlm.nih.gov/) and the dna analytics software (Agilent Technologies). The positions (Mb) indicate the distance from the telomeric end on the short arm of each chromosome. Mb, mega base. [file FEB4-8-1977-s002.pptx]

## Slide 1
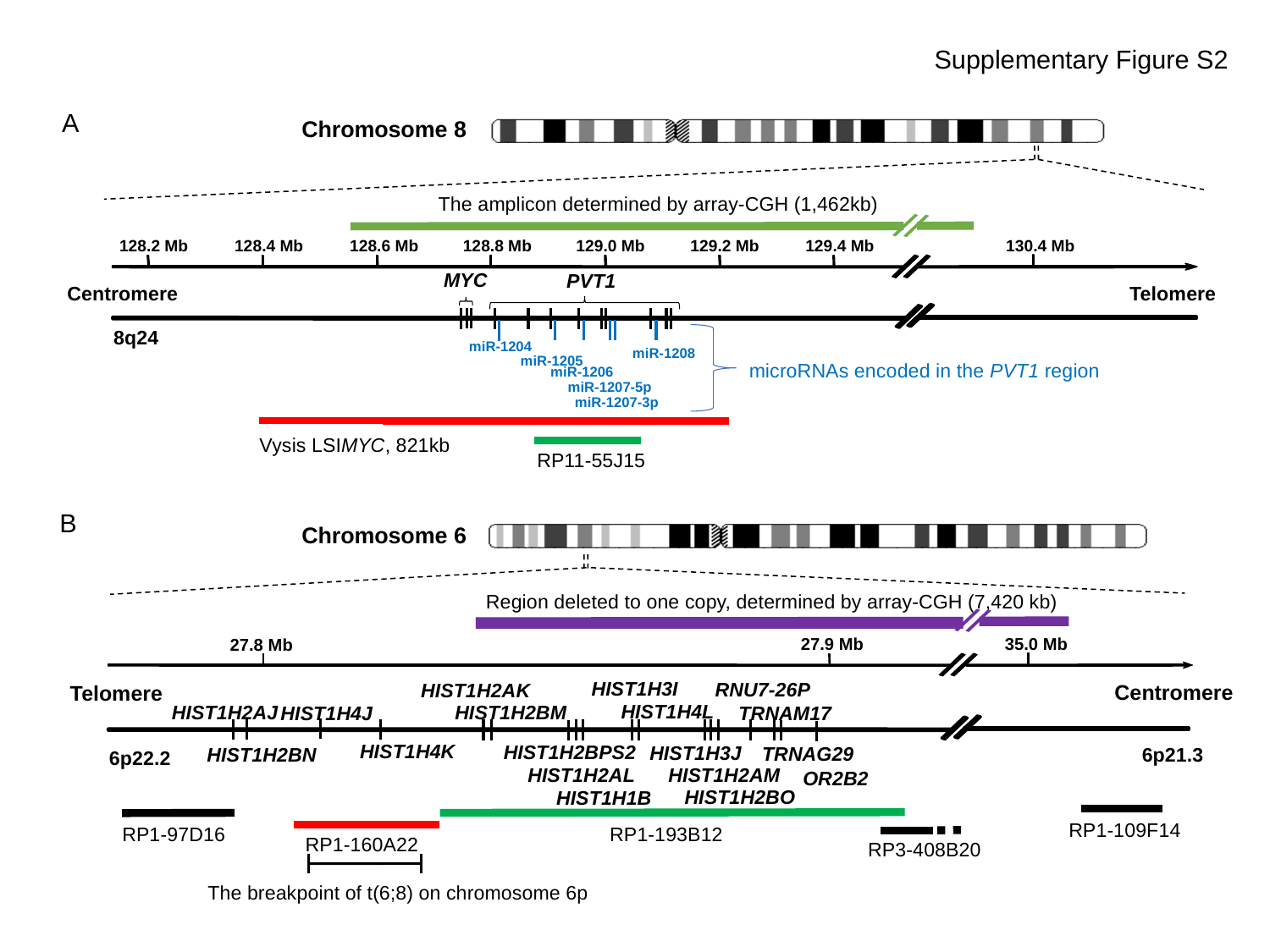

Supplementary Figure S2
A
Chromosome 8
The amplicon determined by array-CGH (1,462kb)
128.8 Mb
129.0 Mb
129.2 Mb
129.4 Mb
130.4 Mb
128.2 Mb
128.4 Mb
128.6 Mb
MYC
PVT1
Centromere
Telomere
8q24
miR-1204
miR-1208
miR-1205
microRNAs encoded in the PVT1 region
miR-1206
miR-1207-5p
miR-1207-3p
Vysis LSIMYC, 821kb
RP11-55J15
B
Chromosome 6
Region deleted to one copy, determined by array-CGH (7,420 kb)
27.9 Mb
35.0 Mb
27.8 Mb
HIST1H3I
RNU7-26P
HIST1H2AK
Centromere
Telomere
HIST1H4L
HIST1H2AJ
HIST1H2BM
HIST1H4J
TRNAM17
HIST1H4K
HIST1H2BPS2
HIST1H3J
TRNAG29
HIST1H2BN
6p21.3
6p22.2
HIST1H2AL
HIST1H2AM
OR2B2
HIST1H2BO
HIST1H1B
RP1-109F14
RP1-193B12
RP1-97D16
RP1-160A22
RP3-408B20
The breakpoint of t(6;8) on chromosome 6p

## Slide 2
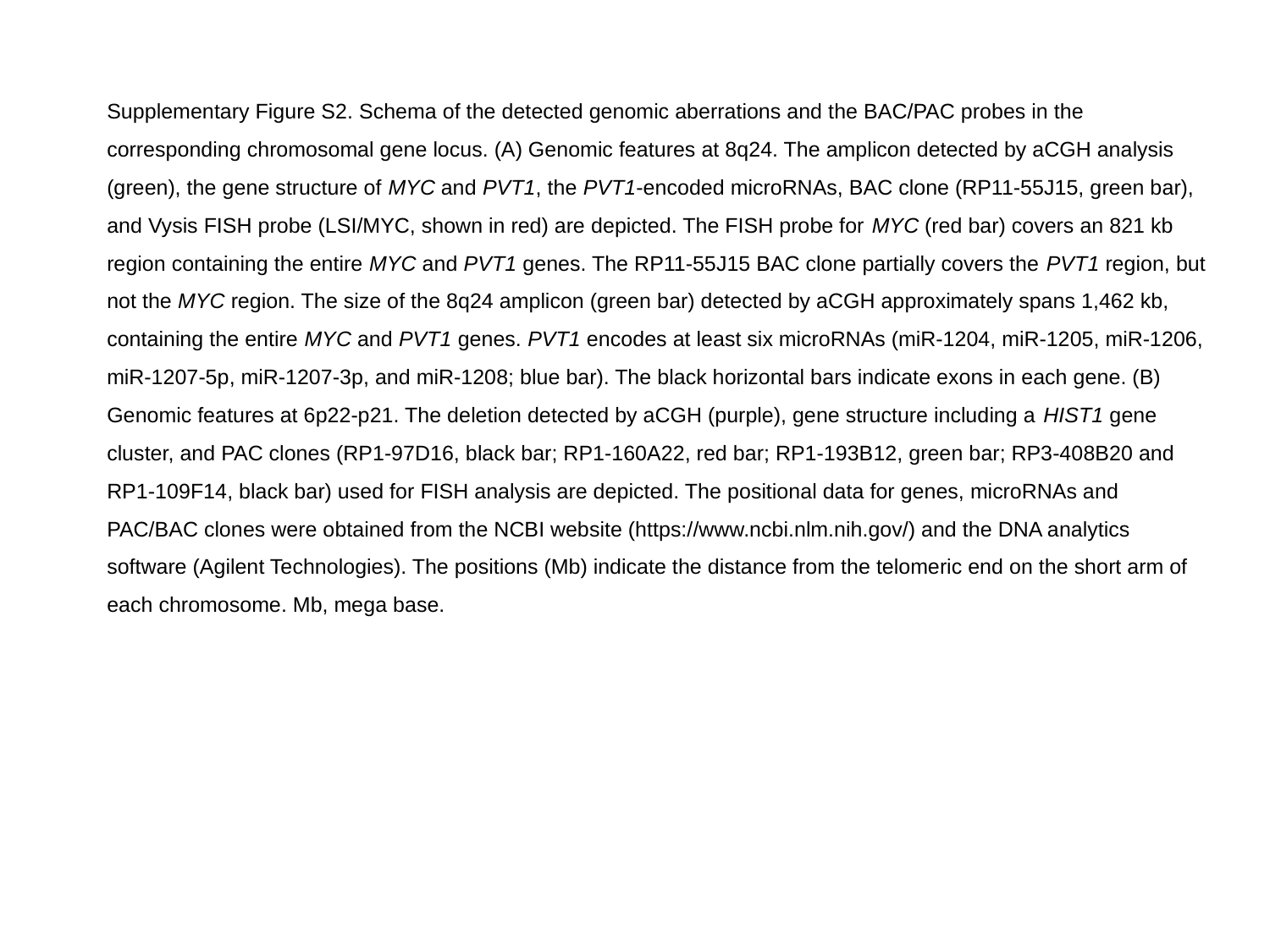

Supplementary Figure S2. Schema of the detected genomic aberrations and the BAC/PAC probes in the corresponding chromosomal gene locus. (A) Genomic features at 8q24. The amplicon detected by aCGH analysis (green), the gene structure of MYC and PVT1, the PVT1-encoded microRNAs, BAC clone (RP11-55J15, green bar), and Vysis FISH probe (LSI/MYC, shown in red) are depicted. The FISH probe for MYC (red bar) covers an 821 kb region containing the entire MYC and PVT1 genes. The RP11-55J15 BAC clone partially covers the PVT1 region, but not the MYC region. The size of the 8q24 amplicon (green bar) detected by aCGH approximately spans 1,462 kb, containing the entire MYC and PVT1 genes. PVT1 encodes at least six microRNAs (miR-1204, miR-1205, miR-1206, miR-1207-5p, miR-1207-3p, and miR-1208; blue bar). The black horizontal bars indicate exons in each gene. (B) Genomic features at 6p22-p21. The deletion detected by aCGH (purple), gene structure including a HIST1 gene cluster, and PAC clones (RP1-97D16, black bar; RP1-160A22, red bar; RP1-193B12, green bar; RP3-408B20 and RP1-109F14, black bar) used for FISH analysis are depicted. The positional data for genes, microRNAs and PAC/BAC clones were obtained from the NCBI website (https://www.ncbi.nlm.nih.gov/) and the DNA analytics software (Agilent Technologies). The positions (Mb) indicate the distance from the telomeric end on the short arm of each chromosome. Mb, mega base.
