# Supplementary material for: Establishment and characterization of a novel vincristine‐resistant diffuse large B‐cell lymphoma cell line containing the 8q24 homogeneously staining region
Source: FEBS Open Bio. 2018 Nov 20;8(12):1977–91. doi: 10.1002/2211-5463.12538 (PMC6275272; doi:10.1002/2211-5463.12538)
Supplement: Supplementary file 3 — Fig. S3. Results of Panther Classification Analysis. Gene ontology analyses using the Panther Classification System. The downregulated genes in cells expressing MYCsh were classified using PANTHER‐Gene List Analysis (http://www.pantherdb.org). The percentages of genes classified into each pathway are shown as a pie chart. [file FEB4-8-1977-s003.pptx]

## Slide 1
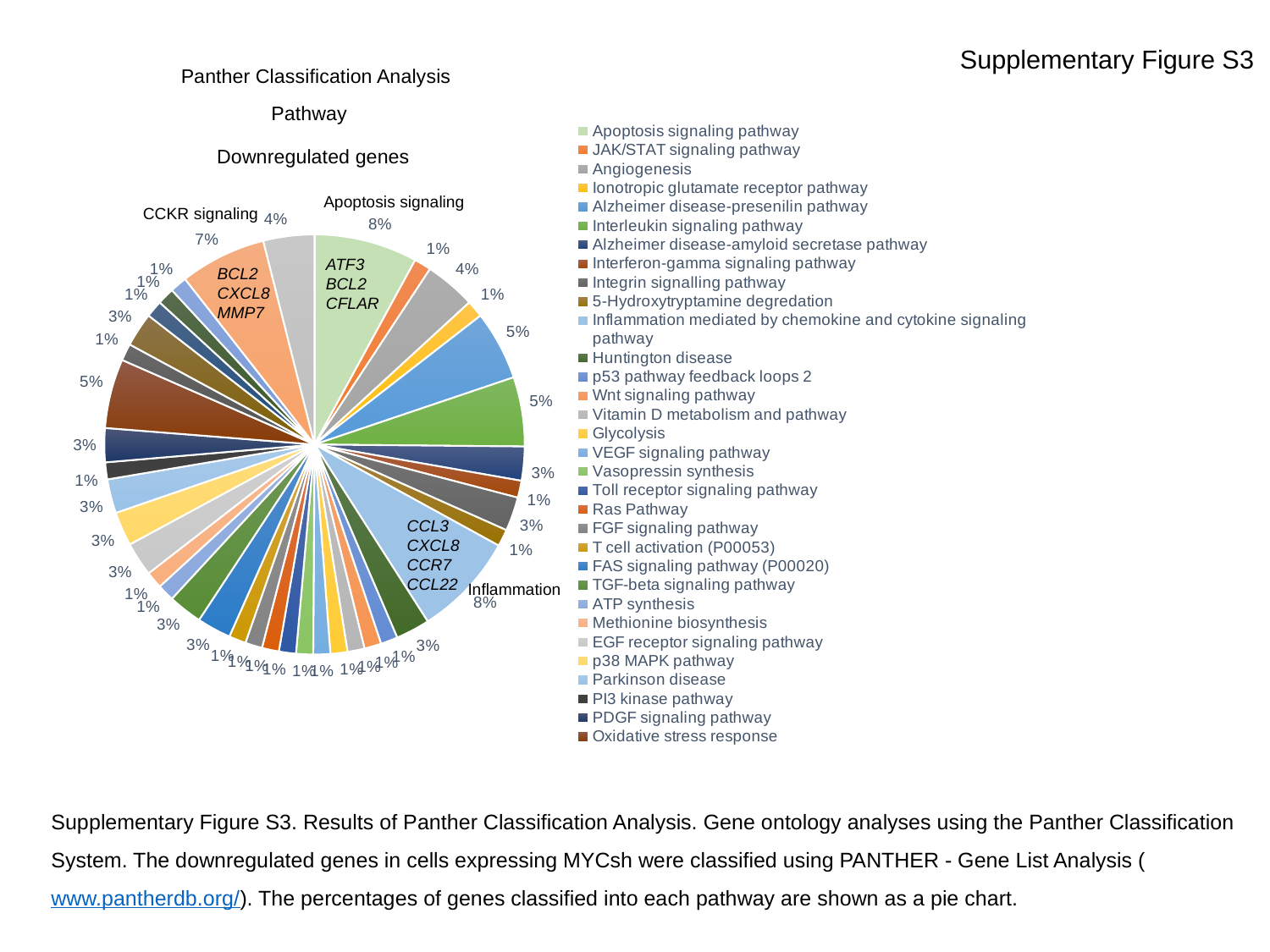

Supplementary Figure S3
Panther Classification Analysis
Pathway
### Chart
| Category | |
|---|---|
| Apoptosis signaling pathway | 0.07900000000000001 |
| JAK/STAT signaling pathway | 0.012999999999999998 |
| Angiogenesis | 0.03900000000000001 |
| Ionotropic glutamate receptor pathway | 0.012999999999999998 |
| Alzheimer disease-presenilin pathway | 0.053000000000000005 |
| Interleukin signaling pathway | 0.053000000000000005 |
| Alzheimer disease-amyloid secretase pathway | 0.026 |
| Interferon-gamma signaling pathway | 0.012999999999999998 |
| Integrin signalling pathway | 0.026 |
| 5-Hydroxytryptamine degredation | 0.012999999999999998 |
| Inflammation mediated by chemokine and cytokine signaling pathway | 0.07900000000000001 |
| Huntington disease | 0.026 |
| p53 pathway feedback loops 2 | 0.012999999999999998 |
| Wnt signaling pathway | 0.012999999999999998 |
| Vitamin D metabolism and pathway | 0.012999999999999998 |
| Glycolysis | 0.012999999999999998 |
| VEGF signaling pathway | 0.012999999999999998 |
| Vasopressin synthesis | 0.012999999999999998 |
| Toll receptor signaling pathway | 0.012999999999999998 |
| Ras Pathway | 0.012999999999999998 |
| FGF signaling pathway | 0.012999999999999998 |
| T cell activation (P00053) | 0.012999999999999998 |
| FAS signaling pathway (P00020) | 0.026 |
| TGF-beta signaling pathway | 0.026 |
| ATP synthesis | 0.012999999999999998 |
| Methionine biosynthesis | 0.012999999999999998 |
| EGF receptor signaling pathway | 0.026 |
| p38 MAPK pathway | 0.026 |
| Parkinson disease | 0.026 |
| PI3 kinase pathway | 0.012999999999999998 |
| PDGF signaling pathway | 0.026 |
| Oxidative stress response | 0.053000000000000005 |
| Cell cycle | 0.012999999999999998 |
| Notch signaling pathway | 0.026 |
| Nicotinic acetylcholine receptor signaling pathway | 0.012999999999999998 |
| Muscarinic acetylcholine receptor 2 and 4 signaling pathway | 0.012999999999999998 |
| B cell activation | 0.012999999999999998 |
| CCKR signaling map | 0.066 |
| Gonadotropin-releasing hormone receptor pathway | 0.03900000000000001 |Downregulated genes
Apoptosis signaling
CCKR signaling
ATF3
BCL2
CFLAR
BCL2
CXCL8
MMP7
CCL3
CXCL8
CCR7
CCL22
Inflammation
Supplementary Figure S3. Results of Panther Classification Analysis. Gene ontology analyses using the Panther Classification System. The downregulated genes in cells expressing MYCsh were classified using PANTHER - Gene List Analysis (www.pantherdb.org/). The percentages of genes classified into each pathway are shown as a pie chart.
