# Supplementary material for: Establishment and characterization of a novel vincristine‐resistant diffuse large B‐cell lymphoma cell line containing the 8q24 homogeneously staining region
Source: FEBS Open Bio. 2018 Nov 20;8(12):1977–91. doi: 10.1002/2211-5463.12538 (PMC6275272; doi:10.1002/2211-5463.12538)
Supplement: Supplementary file 4 — Fig. S4. GSEA with Kyoto Encyclopedia of Genes and Genomes (KEGG) gene sets. GSEA was conducted using GSEA v2.2.4 software and the Molecular Signatures Database (Broad Institute). All of the raw data were formatted and applied to the KEGG gene sets (C2). [file FEB4-8-1977-s004.pptx]

## Slide 1
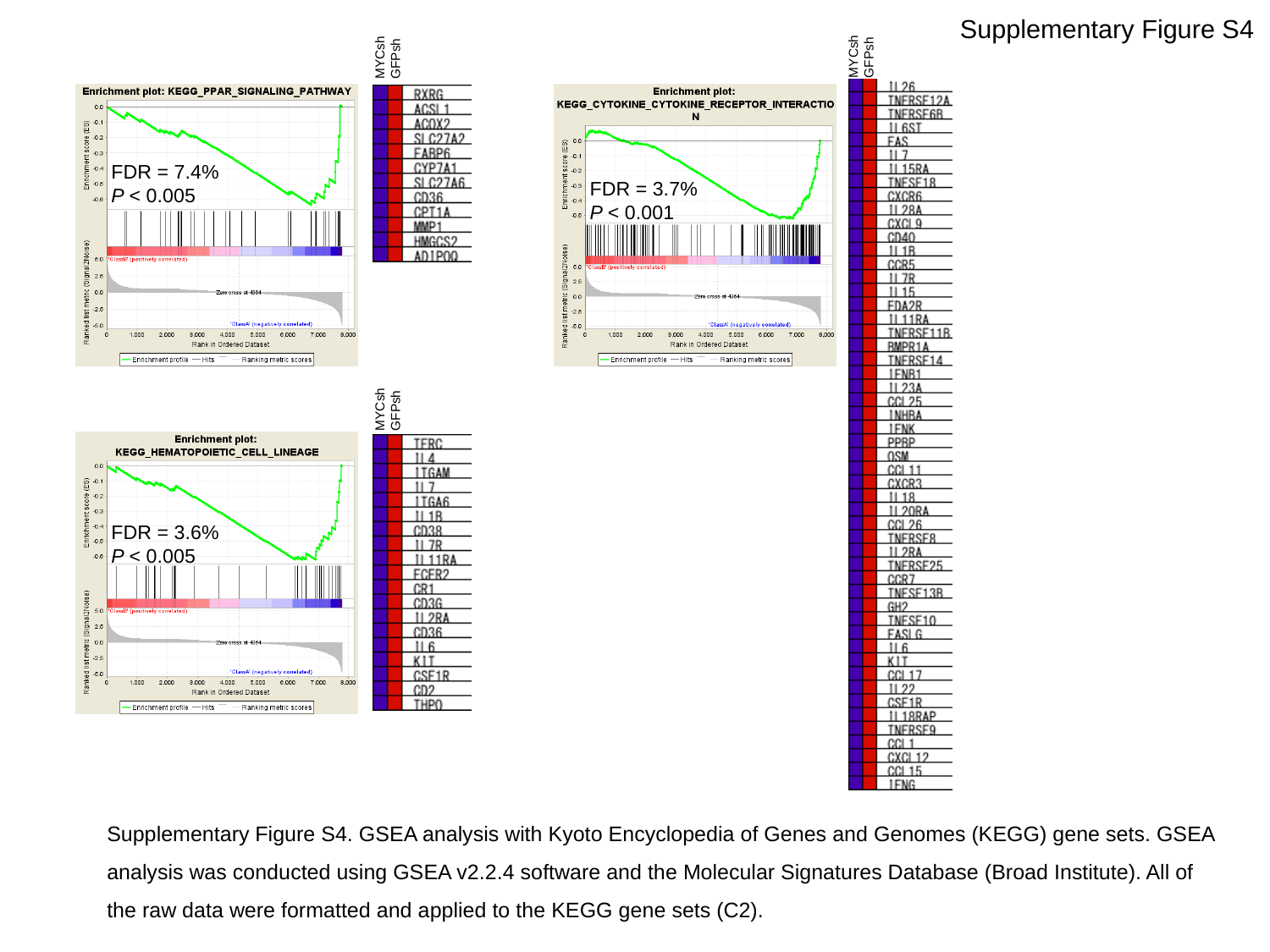

Supplementary Figure S4
MYCsh
GFPsh
MYCsh
GFPsh
FDR = 7.4%
P < 0.005
FDR = 3.7%
P < 0.001
MYCsh
GFPsh
FDR = 3.6%
P < 0.005
Supplementary Figure S4. GSEA analysis with Kyoto Encyclopedia of Genes and Genomes (KEGG) gene sets. GSEA analysis was conducted using GSEA v2.2.4 software and the Molecular Signatures Database (Broad Institute). All of the raw data were formatted and applied to the KEGG gene sets (C2).
