# Supplementary material for: Establishment and characterization of a novel vincristine‐resistant diffuse large B‐cell lymphoma cell line containing the 8q24 homogeneously staining region
Source: FEBS Open Bio. 2018 Nov 20;8(12):1977–91. doi: 10.1002/2211-5463.12538 (PMC6275272; doi:10.1002/2211-5463.12538)
Supplement: Supplementary file 5 — Fig. S5. Sequencing analysis of TP53 gene in AMU‐ML2 cells. (A) Total RNA was isolated from AMU‐ML2 cells using the NucleoSpin RNA kit (TaKaRa Bio, Inc.). After synthesizing complementary DNA, PCR amplification of TP53 gene was performed with a gene‐specific primer set, as described in Online Supplementary Data. Sequence analysis was performed by using an Applied Biosystems 3130 Genetic Analyzer. The TP53 frameshift mutation c.377_378delAC was detected in AMU‐ML2 cells (arrowhead). (B) Sequence alignment of TP53 with wild‐type (WT) TP53 gene. Nucleotide number is in reference to GenBank accession NM_000546.5 (TP53 transcript variant 1, mRNA). [file FEB4-8-1977-s005.pptx]

## Slide 1
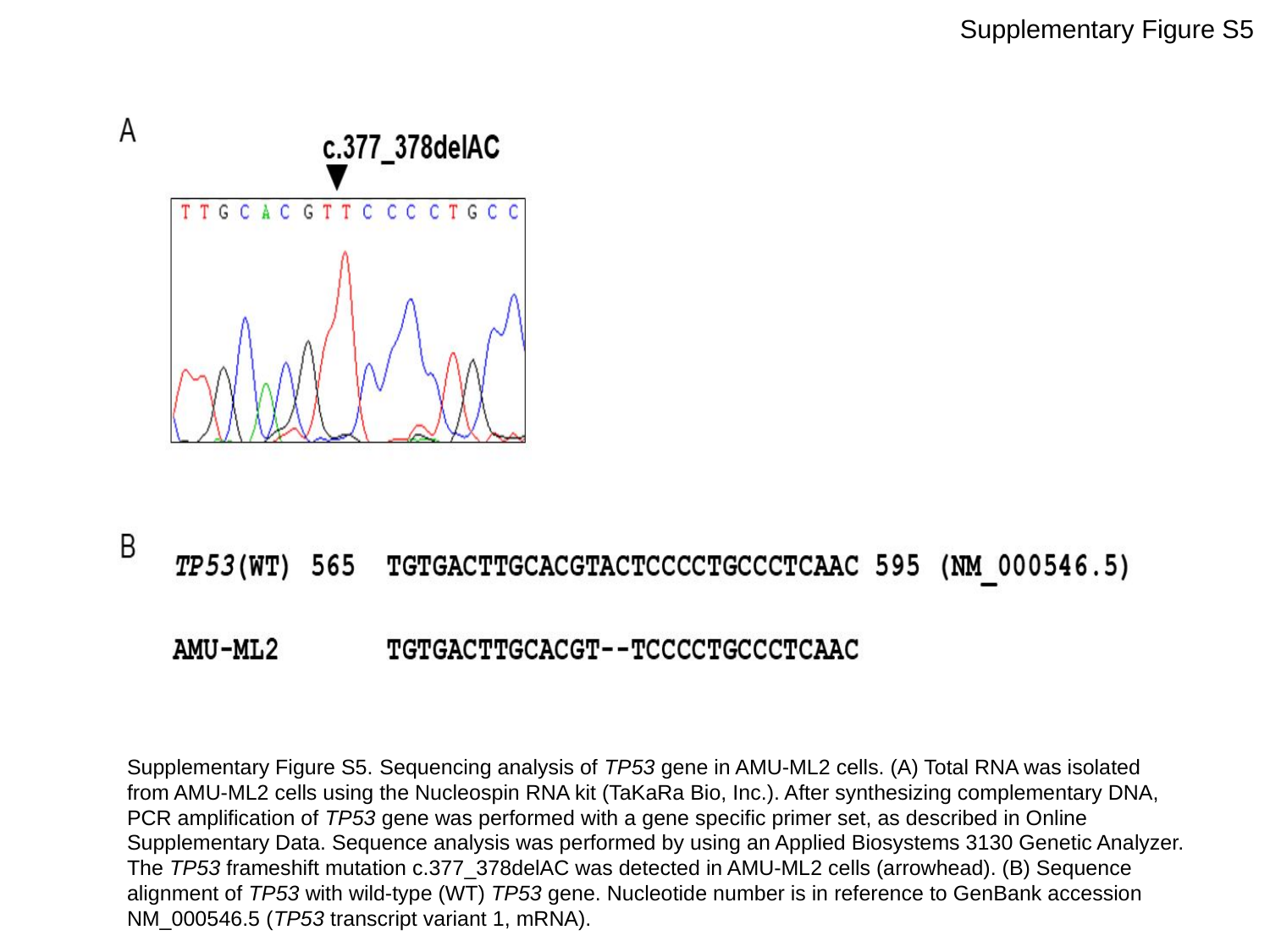

Supplementary Figure S5
Supplementary Figure S5. Sequencing analysis of TP53 gene in AMU-ML2 cells. (A) Total RNA was isolated from AMU-ML2 cells using the Nucleospin RNA kit (TaKaRa Bio, Inc.). After synthesizing complementary DNA, PCR amplification of TP53 gene was performed with a gene specific primer set, as described in Online Supplementary Data. Sequence analysis was performed by using an Applied Biosystems 3130 Genetic Analyzer. The TP53 frameshift mutation c.377_378delAC was detected in AMU-ML2 cells (arrowhead). (B) Sequence alignment of TP53 with wild-type (WT) TP53 gene. Nucleotide number is in reference to GenBank accession NM_000546.5 (TP53 transcript variant 1, mRNA).
