# Supplementary material for: Establishment and characterization of a novel vincristine‐resistant diffuse large B‐cell lymphoma cell line containing the 8q24 homogeneously staining region
Source: FEBS Open Bio. 2018 Nov 20;8(12):1977–91. doi: 10.1002/2211-5463.12538 (PMC6275272; doi:10.1002/2211-5463.12538)
Supplement: Supplementary file 6 — Table S1. Downregulated genes under MYC knockdown in AMU‐ML2 cells. Table S2. Upregulated genes under MYC knockdown in AMU‐ML2 cells. [file FEB4-8-1977-s006.docx]

| Supplementary Table S1. Downregulated genes under *MYC* knockdown in AMU-ML2 cells | | | |
| --- | --- | --- | --- |
| ProbeName | GeneName | Description | Fold change |
| A_33_P3399064 | *RNA5-8S5* | 5.8S ribosomal 5, ribosomal RNA | 0.11 |
| A_23_P317244 | *SPACA3* | sperm acrosome associated 3 | 0.13 |
| A_23_P114903 | *HSPA6* | heat shock 70kDa protein 6 | 0.14 |
| A_33_P3338121 | *LAMB3* | laminin, beta 3 | 0.16 |
| A_24_P313418 | *CCL22* | chemokine (C-C motif) ligand 22 | 0.17 |
| A_23_P29773 | *LAMP3* | lysosomal-associated membrane protein 3 | 0.18 |
| A_23_P62115 | *TIMP1* | TIMP metallopeptidase inhibitor 1 | 0.19 |
| A_21_P0012042 | *ENST00000429730* | Unknown | 0.20 |
| A_33_P3423941 | *IFITM1* | interferon induced transmembrane protein 1 | 0.20 |
| A_23_P131676 | *ACKR3* | atypical chemokine receptor 3 | 0.20 |
| A_24_P176714 | *B9D1* | B9 protein domain 1, variant 2 | 0.23 |
| A_23_P51936 | *TNFRSF9* | tumor necrosis factor receptor superfamily, member 9 | 0.23 |
| A_23_P85693 | *GBP2* | guanylate binding protein 2, interferon-inducible | 0.25 |
| A_23_P413641 | *PREX1* | phosphatidylinositol-3,4,5-trisphosphate-dependent Rac exchange factor 1 | 0.25 |
| A_33_P3415430 | *HSPA1B* | heat shock 70kDa protein 1B | 0.26 |
| A_23_P131024 | *ZBTB32* | zinc finger and BTB domain containing 32 | 0.26 |
| A_23_P147388 | *KIF13B* | kinesin family member 13B | 0.26 |
| A_19_P00317052 | *RNF213* | ring finger protein 213, variant 3 | 0.27 |
| A_24_P278747 | *CCND2* | cyclin D2 | 0.27 |
| A_23_P418031 | *IFFO2* | intermediate filament family orphan 2 | 0.27 |
| A_21_P0000224 | *SNORD83A* | small nucleolar RNA, C/D box 83A | 0.27 |
| A_23_P16252 | *KLK1* | kallikrein 1 | 0.27 |
| A_32_P452655 | *LGALS9C* | lectin, galactoside-binding, soluble, 9C | 0.28 |
| A_33_P3294509 | *CD44* | CD44 molecule | 0.28 |
| A_23_P141651 | *NCOR1* | nuclear receptor corepressor 1 | 0.29 |
| A_23_P52761 | *MMP7* | matrix metallopeptidase 7 | 0.29 |
| A_24_P203000 | *IL2RB* | interleukin 2 receptor, beta | 0.29 |
| A_23_P71148 | *BLVRA* | biliverdin reductase A | 0.29 |
| A_23_P26325 | *CCL17* | chemokine (C-C motif) ligand 17 | 0.29 |
| A_33_P3369716 | *LOC100507195* | uncharacterized LOC100507195, long non-coding RNA | 0.29 |
| A_24_P270460 | *IFI27* | interferon, alpha-inducible protein 27, variant 2 | 0.30 |
| A_23_P201459 | *IFI6* | interferon, alpha-inducible protein 6, variant 3 | 0.30 |
| A_21_P0001246 | *LOC101927851* | Unknown | 0.30 |
| A_23_P110791 | *CSF1R* | colony stimulating factor 1 receptor | 0.30 |
| A_33_P3268567 | *NCK2* | NCK adaptor protein 2 | 0.30 |
| A_21_P0007070 | *lnc-RPP30-2* | lincRNA, lnc-RPP30-2 | 0.31 |
| A_33_P3293207 | *C8orf37* | chromosome 8 open reading frame 37 | 0.31 |
| A_23_P139786 | *OASL* | 2'-5'-oligoadenylate synthetase-like | 0.31 |
| A_23_P85716 | *FCGR2A* | Fc fragment of IgG, low affinity IIa, receptor (CD32) | 0.31 |
| A_23_P208293 | *PVRL2* | poliovirus receptor-related 2 | 0.32 |
| A_23_P138435 | *ZMIZ1* | zinc finger, MIZ-type containing 1 | 0.32 |
| A_33_P3226810 | *TNFSF10* | tumor necrosis factor (ligand) superfamily, member 10 | 0.33 |
| A_33_P3342081 | *PRDM1* | PR domain containing 1, with ZNF domain | 0.33 |
| A_33_P3303414 | *MAN1A1* | mannosidase, alpha, class 1A, member 1 | 0.34 |
| A_23_P34915 | *ATF3* | activating transcription factor 3, variant 4 | 0.34 |
| A_23_P141394 | *WIPI1* | WD repeat domain, phosphoinositide interacting 1 | 0.34 |
| A_33_P3271051 | *CYTH3* | cytohesin 3 | 0.34 |
| A_23_P214360 | *IRF4* | interferon regulatory factor 4 | 0.35 |
| A_23_P119042 | *NKG7* | natural killer cell granule protein 7 | 0.35 |
| A_33_P3383029 | *MXI1* | MAX interactor 1, dimerization protein, variant 2 | 0.35 |
| A_23_P86653 | *SRGN* | serglycin | 0.35 |
| A_24_P230563 | *IL2RA* | interleukin 2 receptor, alpha | 0.36 |
| A_23_P334173 | *LY75* | lymphocyte antigen 75 | 0.36 |
| A_21_P0010689 | *XLOC_l2_000018* | BROAD Institute lincRNA,　XLOC_l2_000018 | 0.36 |
| A_23_P45871 | *IFI44L* | interferon-induced protein 44-like | 0.36 |
| A_23_P62932 | *ATP1B1* | ATPase, Na+/K+ transporting, beta 1 polypeptide | 0.36 |
| A_23_P3221 | *SQRDL* | sulfide quinone reductase-like | 0.36 |
| A_33_P3355185 | *BCL2* | B-cell CLL/lymphoma 2 | 0.37 |
| A_33_P3343485 | *HIP1* | huntingtin interacting protein 1 | 0.37 |
| A_23_P338479 | *CD274* | CD274 molecule (PD-L1) | 0.37 |
| A_33_P3411075 | *FSCN1* | fascin actin-bundling protein 1 | 0.37 |
| A_33_P3226202 | *FAM179B* | family with sequence similarity 179, member B | 0.37 |
| A_23_P215479 | *CLIP2* | CAP-GLY domain containing linker protein 2 | 0.37 |
| A_33_P3354607 | *CCL4L2* | chemokine (C-C motif) ligand 4-like 2 | 0.37 |
| A_33_P3371663 | *LTK* | leukocyte receptor tyrosine kinase | 0.38 |
| A_23_P67529 | *KCNN4* | potassium channel, calcium activated intermediate/small conductance subfamily N | 0.38 |
| A_33_P3400273 | *SELL* | selectin L | 0.38 |
| A_32_P87013 | *CXCL8* | chemokine (C-X-C motif) ligand 8 | 0.38 |
| A_23_P343398 | *CCR7* | chemokine (C-C motif) receptor 7 | 0.38 |
| A_33_P3283611 | *IFIT3* | interferon-induced protein with tetratricopeptide repeats 3, variant 3 | 0.39 |
| A_23_P1962 | *RARRES3* | retinoic acid receptor responder (tazarotene induced) 3 | 0.39 |
| A_33_P3388618 | *TNK1* | tyrosine kinase, non-receptor, 1 | 0.39 |
| A_23_P3532 | *LITAF* | lipopolysaccharide-induced TNF factor | 0.39 |
| A_32_P5480 | *CERS6* | ceramide synthase 6, variant 2 | 0.39 |
| A_23_P153026 | *GAA* | glucosidase, alpha; acid | 0.39 |
| A_23_P42257 | *IER3* | immediate early response 3 | 0.39 |
| A_23_P132159 | *USP18* | ubiquitin specific peptidase 18 | 0.39 |
| A_24_P157926 | *TNFAIP3* | tumor necrosis factor, alpha-induced protein 3, variant 3 | 0.39 |
| A_23_P41470 | *DDX60* | DEAD (Asp-Glu-Ala-Asp) box polypeptide 60 | 0.40 |
| A_33_P3376971 | *CHAC1* | ChaC glutathione-specific gamma-glutamylcyclotransferase 1 | 0.40 |
| A_33_P3271111 | *NINJ1* | ninjurin 1 | 0.40 |
| A_23_P133691 | *RRAGD* | Ras-related GTP binding D | 0.40 |
| A_23_P389250 | *SMCO2* | single-pass membrane protein with coiled-coil domains 2 | 0.40 |
| A_24_P406132 | *MAPK13* | mitogen-activated protein kinase 13 | 0.40 |
| A_23_P68031 | *STAT4* | signal transducer and activator of transcription 4 | 0.41 |
| A_24_P45446 | *GBP4* | guanylate binding protein 4 | 0.41 |
| A_23_P103398 | *PSEN2* | presenilin 2 | 0.41 |
| A_23_P209394 | *CFLAR* | CASP8 and FADD-like apoptosis regulator | 0.42 |
| A_23_P55518 | *SMAD7* | SMAD family member 7 | 0.42 |
| A_23_P22499 | *GNL3L* | guanine nucleotide binding protein-like 3-like, variant 2 | 0.42 |
| A_23_P500614 | *TNFRSF8* | tumor necrosis factor receptor superfamily, member 8 | 0.42 |
| A_33_P3218980 | *ENTPD1* | ectonucleoside triphosphate diphosphohydrolase 1 | 0.42 |
| A_21_P0000492 | *SNAR-F* | small ILF3/NF90-associated RNA F | 0.42 |
| A_24_P941167 | *APOL6* | apolipoprotein L, 6 | 0.42 |
| A_24_P236091 | *ENO2* | enolase 2, gamma, neuronal | 0.42 |
| A_23_P90944 | *SCN7A* | cancer susceptibility candidate 15, long non-coding RNA | 0.42 |
| A_23_P52266 | *IFIT1* | interferon-induced protein with tetratricopeptide repeats 1 | 0.42 |
| A_23_P68155 | *IFIH1* | interferon induced with helicase C domain 1 | 0.42 |
| A_19_P00325158 | *MALAT1* | metastasis associated lung adenocarcinoma transcript 1 | 0.43 |
| A_23_P301925 | *COX1* | mitochondrially encoded cytochrome c oxidase I | 0.43 |
| A_33_P3405424 | *IL4I1* | interleukin 4 induced 1 | 0.43 |
| A_21_P0010193 | *lnc-MRPL39-4* | lincRNA (lnc-MRPL39-4) | 0.43 |
| A_23_P343411 | *AGRN* | agrin | 0.43 |
| A_33_P3264895 | *RHEBL1* | Ras homolog enriched in brain like 1 | 0.43 |
| A_23_P24716 | *TMEM132A* | transmembrane protein 132A | 0.43 |
| A_21_P0000385 | *SNORD88C* | small nucleolar RNA, C/D box 88C | 0.44 |
| A_23_P319617 | *CHST7* | carbohydrate (N-acetylglucosamine 6-O) sulfotransferase 7 | 0.44 |
| A_23_P143016 | *ARID5A* | AT rich interactive domain 5A | 0.44 |
| A_32_P167076 | *CAPN14* | uncharacterized LOC400958 | 0.44 |
| A_23_P125078 | *SLC26A11* | solute carrier family 26 (anion exchanger), member 11, variant 2 | 0.44 |
| A_33_P3386547 | *SGPP2* | sphingosine-1-phosphate phosphatase 2 | 0.44 |
| A_23_P90419 | *PBX4* | pre-B-cell leukemia homeobox 4 | 0.44 |
| A_33_P3413558 | *CD226* | CD226 molecule | 0.44 |
| A_32_P197340 | *ERICH2* | glutamate-rich 2 | 0.44 |
| A_23_P166459 | *LGALS1* | lectin, galactoside-binding, soluble, 1 | 0.44 |
| A_23_P250619 | *ZDHHC14* | zinc finger, DHHC-type containing 14 | 0.45 |
| A_24_P288993 | *ENST00000513465* | Unknown | 0.45 |
| A_23_P134935 | *DUSP4* | dual specificity phosphatase 4 | 0.45 |
| A_21_P0013394 | *LOC102723946* | Unknown | 0.45 |
| A_23_P142075 | *ACP5* | acid phosphatase 5, tartrate resistant , variant 4 | 0.45 |
| A_33_P3316273 | *CCL3* | chemokine (C-C motif) ligand 3 | 0.45 |
| A_23_P160720 | *BATF3* | basic leucine zipper transcription factor, ATF-like 3 | 0.45 |
| A_23_P321201 | *DENND5A* | DENN/MADD domain containing 5A | 0.45 |
| A_33_P3229196 | *CD151* | CD151 molecule | 0.45 |
| A_21_P0010921 | *GLUD1P7* | glutamate dehydrogenase 1 pseudogene 7 | 0.46 |
| A_24_P28722 | *RSAD2* | radical S-adenosyl methionine domain containing 2 | 0.46 |
| A_23_P70318 | *ENPP4* | ectonucleotide pyrophosphatase/phosphodiesterase 4 | 0.46 |
| A_24_P353638 | *SLAMF7* | SLAM family member 7 | 0.46 |
| A_23_P34835 | *LMNA* | lamin A/C, varient 2 | 0.46 |
| A_24_P53051 | *LACTB* | lactamase, beta | 0.46 |
| A_23_P356616 | *ABTB2* | ankyrin repeat and BTB (POZ) domain containing 2 | 0.46 |
| A_24_P324405 | *ANKRD11* | ankyrin repeat domain 11, variant 2 | 0.46 |
| A_33_P3362088 | *P2RX4* | purinergic receptor P2X, ligand gated ion channel, 4 | 0.47 |
| A_23_P200792 | *NOTCH2* | notch 2 | 0.47 |
| A_24_P228130 | *CCL3L3* | chemokine (C-C motif) ligand 3-like 3 | 0.47 |
| A_33_P3272921 | *ARID3A* | AT rich interactive domain 3A | 0.47 |
| A_32_P214925 | *TCAF2* | family with sequence similarity 115, member C, variant 2 | 0.47 |
| A_24_P319364 | *F11R* | F11 receptor | 0.47 |
| A_23_P46369 | *RAB13* | RAB13, member RAS oncogene family | 0.47 |
| A_23_P119907 | *ANKZF1* | ankyrin repeat and zinc finger domain containing 1 | 0.47 |
| A_32_P166693 | *HEG1* | heart development protein with EGF-like domains 1 | 0.47 |
| A_23_P132057 | *LINC00158* | long intergenic non-protein coding RNA 158 | 0.48 |
| A_33_P3225512 | *OAS2* | 2'-5'-oligoadenylate synthetase 2, 69/71kDa, variant 2 | 0.48 |
| A_23_P126103 | *CTH* | cystathionine gamma-lyase | 0.48 |
| A_33_P3718269 | *MIR146A* | microRNA 146a | 0.48 |
| A_23_P41246 | *MFSD10* | major facilitator superfamily domain containing 10 | 0.48 |
| A_23_P119478 | *EBI3* | Epstein-Barr virus induced 3 | 0.48 |
| A_32_P129950 | *NHLRC3* | NHL repeat containing 3 | 0.48 |
| A_33_P3424577 | *ENST00000613594* | T cell receptor beta constant 1 | 0.48 |
| A_21_P0012252 | *ENST00000410028* | tubulin, alpha 3g, pseudogene | 0.48 |
| A_23_P134113 | *SLC18B1* | solute carrier family 18, subfamily B, member 1 | 0.48 |
| A_33_P3414880 | *LOC339192* | uncharacterized LOC339192 | 0.48 |
| A_33_P3418170 | *DDX58* | DEAD (Asp-Glu-Ala-Asp) box polypeptide 58 | 0.48 |
| A_33_P3352827 | *SLAMF1* | signaling lymphocytic activation molecule family member 1 | 0.48 |
| A_33_P3281985 | *CR2* | complement component receptor 2 (CR2) | 0.48 |
| A_23_P156788 | *STX11* | syntaxin 11 | 0.49 |
| A_21_P0013662 | *HSPB1* | heat shock 27kDa protein 1 | 0.49 |
| A_33_P3364864 | *NAMPT* | nicotinamide phosphoribosyltransferase | 0.49 |
| A_23_P102950 | *RSPH1* | radial spoke head 1 homolog | 0.49 |
| A_24_P295590 | *RASSF4* | Ras association domain family member 4 | 0.49 |
| A_24_P37409 | *DUSP2* | dual specificity phosphatase 2 | 0.49 |
| A_23_P106389 | *SEMA7A* | semaphorin 7A, GPI membrane anchor | 0.49 |
| A_24_P216253 | *DLGAP4* | discs, large homolog-associated protein 4 | 0.49 |
| A_23_P162589 | *VDR* | vitamin D (1,25- dihydroxyvitamin D3) receptor, variant 2 | 0.49 |
| A_23_P214222 | *MARCKS* | myristoylated alanine-rich protein kinase C substrate | 0.50 |
| A_32_P108156 | *MIR155HG* | MIR155 host gene | 0.50 |
| A_33_P3257182 | *MYBPC2* | myosin binding protein C, fast type | 0.50 |
| A_23_P408353 | *HLA-A* | major histocompatibility complex, class I, A | 0.50 |
| A_33_P3260654 | *ENST00000610439* | T cell receptor beta constant 1 | 0.50 |
| A_23_P88580 | *ARID3B* | AT rich interactive domain 3B | 0.50 |
| A_33_P3240674 | *BRD3* | bromodomain containing 3 | 0.50 |
| A_33_P3231414 | *LILRB1* | leukocyte immunoglobulin-like receptor, subfamily B, member 1 | 0.50 |

| Supplementary Table S2. Upregulated genes under *MYC* knockdown in AMU-ML2 cells | | | |
| --- | --- | --- | --- |
| ProbeName | GeneName | Description | Fold change |
| A_23_P167129 | *HHIP* | hedgehog interacting protein | 3791.6 |
| A_33_P3344339 | *KCNH5* | potassium channel, voltage gated eag related subfamily H, member 5 | 207.2 |
| A_23_P69329 | *HYAL1* | DDB1 and CUL4 associated factor 10 | 158.6 |
| A_23_P27353 | *SLC14A2* | lnc-GABARAPL1-1, lincRNA | 148.1 |
| A_33_P3334630 | *PLP1* | proteolipid protein 1 | 136.3 |
| A_21_P0008780 | *lnc-VPS33B-2* | lnc-VPS33B-2, lincRNA | 132.6 |
| A_23_P154806 | *EPB41L1* | erythrocyte membrane protein band 4.1-like 1 | 123.5 |
| A_21_P0003972 | *lnc-FBXL7-3* | clone IMAGE:5538207, mRNA | 107.0 |
| A_23_P371758 | *SDR9C7* | short chain dehydrogenase/reductase family 9C, member 7 | 99.3 |
| A_23_P425990 | *MTUS2* | microtubule associated tumor suppressor candidate 2 | 93.6 |
| A_21_P0007669 | *lnc-FZD10-3* | lnc-ZNF366-2, lincRNA | 93.3 |
| A_33_P3260680 | *OR2T4* | olfactory receptor, family 2, subfamily T, member 4 | 91.7 |
| A_32_P117313 | *TRIQK* | triple QxxK/R motif containing | 91.5 |
| A_33_P3218380 | *LOC100129345* | LOC100129345, long non-coding RNA | 87.2 |
| A_33_P3385376 | *SYPL2* | synaptophysin-like 2 | 84.7 |
| A_33_P3209541 | *KIAA1875* | KIAA1875, long non-coding RNA | 80.3 |
| A_21_P0005131 | *lnc-TRMT11-1* | lnc-TRMT11-1, lincRNA | 77.8 |
| A_23_P106906 | *PPL* | periplakin | 74.1 |
| A_23_P115261 | *AGT* | angiotensinogen | 59.8 |
| A_21_P0012037 | *LOC101927641* | LOC101927641, long non-coding RNA | 57.1 |
| A_33_P3308167 | *POLR2J4* | ankyrin repeat domain 22 | 43.8 |
| A_33_P3253175 | *WDR37* | WD repeat domain 37 | 39.3 |
| A_33_P3249649 | *CACTIN-AS1* | CACTIN antisense RNA 1, long non-coding RNA | 29.3 |
| A_33_P3354783 | *A_33_P3354783* | Unknown | 22.9 |
| A_21_P0014412 | *lnc-GABPA-2* | RST41516 Athersys RAGE Library cDNA | 21.7 |
| A_23_P111662 | *ABCB5* | solute carrier family 23 , member 1 | 17.1 |
| A_33_P3247342 | *ANO7* | anoctamin 7 | 16.5 |
| A_24_P217330 | *POMGNT2* | protein O-linked mannose N-acetylglucosaminyltransferase 2 | 14.7 |
| A_24_P405981 | *TTC33* | tetratricopeptide repeat domain 33 | 14.2 |
| A_23_P108265 | *OR7C2* | olfactory receptor, family 7, subfamily C, member 2 | 11.5 |
| A_33_P3327192 | *ENST00000453179* | BRALZ2 cDNA clone BRALZ2017105 5' | 10.7 |
| A_23_P100963 | *SPNS3* | spinster homolog 3 | 7.6 |
| A_21_P0003499 | *lnc-ANKRD50-2* | CCAAT/enhancer binding protein, delta | 7.4 |
| A_23_P82868 | *PLAT* | CDGSH iron sulfur domain 3 | 7.4 |
| A_21_P0004077 | *ENST00000510150* | tripartite motif containing 46 | 7.3 |
| A_21_P0000504 | *RNU6ATAC* | RNA, U6atac small nuclear, small nuclear RNA | 6.9 |
| A_33_P3308626 | *MON1B* | MON1 secretory trafficking family member B | 6.7 |
| A_24_P313993 | *CAPS* | linc\|LNCipedia lincRNA (lnc-TSC22D1-1), lincRNA [lnc-TSC22D1-1:4] | 6.7 |
| A_23_P115444 | *TNFSF18* | Unknown | 6.6 |
| A_23_P37914 | *SLC5A11* | CDC42 effector protein5 | 6.6 |
| A_33_P3218797 | *PPDPF* | pancreatic progenitor cell differentiation and proliferation factor | 6.2 |
| A_21_P0011811 | *ANKRD36BP2* | ankyrin repeat domain 36B pseudogene, non-coding RNA | 5.9 |
| A_24_P384397 | *RAVER1* | ribonucleoprotein, PTB-binding 1 | 5.9 |
| A_24_P883629 | *C2orf49* | chromosome 2 open reading frame 49 | 5.8 |
| A_24_P203953 | *LOC439951* | LOC439951, misc_RNA | 5.8 |
| A_33_P3392807 | *TAF1B* | TMPRSS4 antisense RNA 1, long non-coding RNA | 5.6 |
| A_23_P256663 | *GALR3* | galanin receptor 3 | 5.5 |
| A_19_P00320434 | *LOC100996579* | LOC100996579, long non-coding RNA | 5.5 |
| A_33_P3312676 | *ENST00000621996* | myelin transcription factor 1 | 5.2 |
| A_33_P3229572 | *RASA4B* | long intergenic non-protein coding RNA 1001 | 5.1 |
| A_33_P3407657 | *MGRN1* | cerebral cavernous malformation 2-like | 5.1 |
| A_33_P3299599 | *NKX2-5* | NK2 homeobox 5 | 5.0 |
| A_23_P146885 | *UTS2R* | urotensin 2 receptor | 5.0 |
| A_33_P3441583 | *BM453041* | cDNA clone IMAGE:5529651 5' | 5.0 |
| A_23_P112874 | *GPC5* | secretoglobin, family 3A, member 1 | 4.9 |
| A_33_P3319937 | *A_33_P3319937* | Unknown | 4.9 |
| A_24_P342312 | *TENM4* | nc-STK35-1, lincRNA | 4.9 |
| A_33_P3306526 | *A_33_P3306526* | Unknown | 4.8 |
| A_33_P3235117 | *LOC100133286* | LOC100133286, long non-coding RNA | 4.7 |
| A_23_P333129 | *ENST00000425409* | double homeobox 4 like 11 | 4.7 |
| A_24_P348083 | *BC093850* | chromosome 18 open reading frame 23 | 4.6 |
| A_33_P3266998 | *FAM106B* | Unknown | 4.6 |
| A_33_P3546363 | *TUSC8* | cDNA clone IMAGE:6199956 5' | 4.4 |
| A_33_P3242578 | *LOC100506731* | Unknown | 4.4 |
| A_33_P3328659 | *CELSR1* | cadherin, EGF LAG seven-pass G-type receptor 1 | 4.4 |
| A_19_P00321628 | *LINC00888* | long intergenic non-protein coding RNA 888 | 4.2 |
| A_33_P3280325 | *A_33_P3280325* | Unknown | 4.2 |
| A_24_P831309 | *C1orf229* | chromosome 1 open reading frame 229 | 4.2 |
| A_33_P3267263 | *RNVU1-18* | RNVU1-18, small nuclear RNA | 4.1 |
| A_23_P161171 | *ASAH2* | zinc finger protein 329 | 4.0 |
| A_23_P7582 | *TCF7* | Unknown | 4.0 |
| A_33_P3407374 | *A_33_P3407374* | Unknown | 3.9 |
| A_23_P140527 | *FOXB1* | forkhead box B1 | 3.9 |
| A_23_P86493 | *LBX1* | ladybird homeobox 1 | 3.9 |
| A_24_P37887 | *GPR150* | G protein-coupled receptor 150 | 3.9 |
| A_23_P64873 | *DCN* | decorin | 3.9 |
| A_21_P0008768 | *LOC101929641* | LOC101929641 | 3.8 |
| A_33_P3474175 | *SFTA1P* | cDNA clone IMAGE:6495000 5' | 3.7 |
| A_33_P3424057 | *PEG3* | small nucleolar RNA host gene 11 | 3.7 |
| A_33_P3410206 | *VPS9D1* | VPS9 domain containing 1 | 3.6 |
| A_33_P3330125 | *DIABLO* | diablo, IAP-binding mitochondrial protein | 3.6 |
| A_32_P150735 | *THC2610134* | cDNA DKFZp547L112 | 3.6 |
| A_23_P72462 | *ATP2A1* | scavenger receptor class F, member 2 | 3.5 |
| A_33_P3312030 | *ENST00000507296* | Unknown | 3.5 |
| A_23_P147918 | *S100A16* | hyperpolarization activated cyclic nucleotide gated potassium channel 2 | 3.5 |
| A_33_P3417176 | *lnc-DTYMK-3* | lnc-DTYMK-3, lincRNA | 3.5 |
| A_33_P3221989 | *CACNB4* | extracellular leucine-rich repeat and fibronectin type III domain containing 1 | 3.4 |
| A_24_P618401 | *CKMT2-AS1* | Sp8 transcription factor | 3.4 |
| A_33_P3340862 | *TMEM88B* | transmembrane protein 88B | 3.4 |
| A_33_P3379039 | *IGLL5* | immunoglobulin lambda-like polypeptide 5 | 3.4 |
| A_23_P28707 | *OGFR* | opioid growth factor receptor | 3.3 |
| A_33_P3334773 | *MYOD1* | transmembrane protein 8C | 3.3 |
| A_23_P165333 | *BIN1* | lnc-TRAPPC12-3, lincRNA | 3.3 |
| A_33_P3381255 | *CLEC2A* | torsin family 2, member A | 3.3 |
| A_33_P3304576 | *KRTAP5-5* | Unknown | 3.3 |
| A_33_P3285715 | *GLI4* | GLI family zinc finger 4 | 3.3 |
| A_19_P00318375 | *LOC148696* | tumor suppressor candidate 1 | 3.3 |
| A_33_P3363290 | *A_33_P3363290* | Rho GTPase activating protein 2 | 3.2 |
| A_32_P124708 | *ONECUT2* | LIM homeobox 3, transcript variant 2 | 3.2 |
| A_23_P47034 | *HHEX* | hematopoietically expressed homeobox | 3.2 |
| A_33_P3220149 | *MAML1* | mastermind-like 1 | 3.2 |
| A_23_P8702 | *PIP* | prolactin-induced protein | 3.1 |
| A_33_P3347971 | *TPD52* | tumor protein D52 | 3.1 |
| A_32_P63562 | *lnc-DNTTIP2-1* | nuclear factor of kappa light polypeptide gene enhancer in B-cells inhibitor-like 1 | 3.1 |
| A_33_P3238548 | *C3orf36* | Ly6/neurotoxin 1 | 3.1 |
| A_23_P14165 | *GPR18* | G protein-coupled receptor 18 | 3.1 |
| A_33_P3437273 | *LOC84843* | long intergenic non-protein coding RNA 1150 | 3.0 |
| A_33_P3410895 | *POR* | P450 (cytochrome) oxidoreductase | 3.0 |
| A_33_P3353906 | *A_33_P3353906* | Unknown | 3.0 |
| A_33_P3370094 | *MME* | membrane metallo-endopeptidase | 3.0 |
| A_33_P3329419 | *DNM1* | dynamin 1 | 3.0 |
| A_33_P3411388 | *UNCX* | UNC homeobox | 3.0 |
| A_23_P104413 | *DUX4* | double homeobox 4 | 3.0 |
| A_32_P222695 | *ARHGEF37* | Rho guanine nucleotide exchange factor 37 | 3.0 |
| A_21_P0014286 | *LINC00456* | cDNA clone IMAGE:5485440 5' | 3.0 |
| A_23_P25674 | *CKB* | long intergenic non-protein coding RNA 176 | 2.9 |
| A_23_P1912 | *ZP1* | Unknown | 2.9 |
| A_23_P112634 | *SMIM14* | small integral membrane protein 14 | 2.8 |
| A_21_P0002358 | *ENST00000414512* | pyrophosphatase (inorganic) 2 | 2.8 |
| A_23_P411188 | *CHRNA10* | cholinergic receptor, nicotinic, alpha 10 | 2.8 |
| A_33_P3313532 | *ANKRD20A1* | cDNA FLJ45052 fis, clone BRAWH3022542 | 2.8 |
| A_21_P0002208 | *lnc-PRKCE-1* | Unknown | 2.8 |
| A_23_P54116 | *DAAM1* | dishevelled associated activator of morphogenesis 1 | 2.8 |
| A_33_P3318646 | *CALY* | calcyon neuron-specific vesicular protein | 2.8 |
| A_33_P3395675 | *ENST00000390323* | immunoglobulin lambda constant 2 | 2.8 |
| A_21_P0013688 | *XLOC_l2_015418* | NFYC antisense RNA 1, long non-coding RNA | 2.7 |
| A_19_P00321339 | *LOC102724384* | Unknown | 2.7 |
| A_23_P157136 | *SCIN* | proprotein convertase subtilisin/kexin type 1 inhibitor | 2.7 |
| A_33_P3354137 | *MAP4* | microtubule-associated protein 4 | 2.7 |
| A_19_P00319181 | *LOC441204* | zinc finger protein 205 | 2.7 |
| A_33_P3323535 | *ENST00000390453* | proline rich 36 \ | 2.7 |
| A_33_P3422298 | *ENST00000410078* | ens\|immunoglobulin lambda constant 6 | 2.7 |
| A_23_P36641 | *AICDA* | activation-induced cytidine deaminase | 2.7 |
| A_33_P3249434 | *PLAA* | phospholipase A2-activating protein | 2.6 |
| A_21_P0007944 | *LINC00383* | coiled-coil domain containing 144A | 2.6 |
| A_23_P77493 | *TUBB3* | tubulin, beta 3 class III | 2.6 |
| A_33_P3279009 | *HMX1* | H6 family homeobox 1 | 2.6 |
| A_23_P163173 | *LTB4R2* | leukotriene B4 receptor 2 | 2.6 |
| A_19_P00327354 | *LINC00963* | golgin A2 pseudogene 7, non-coding RNA | 2.6 |
| A_33_P3372635 | *CSN1S2AP* | NK1 homeobox 2 | 2.6 |
| A_33_P3269854 | *ENST00000436804* | CASK interacting protein 1 | 2.6 |
| A_33_P3248265 | *LTB* | lymphotoxin beta | 2.5 |
| A_21_P0012423 | *XLOC_l2_010056* | proline rich 33 | 2.5 |
| A_21_P0014180 | *GPR146* | Unknown | 2.5 |
| A_24_P89835 | *MAPK15* | ADAMTS-like 5 | 2.5 |
| A_23_P216108 | *ANK1* | ankyrin 1, erythrocytic | 2.5 |
| A_33_P3369844 | *CD24* | CD24 molecule | 2.5 |
| A_21_P0009322 | *lnc-NEUROD2-1* | Unknown | 2.5 |
| A_21_P0000502 | *RNU11* | RNA, U11 small nuclear | 2.5 |
| A_23_P43157 | *MYBL1* | v-myb avian myeloblastosis viral oncogene homolog-like 1 | 2.5 |
| A_33_P3313411 | *ARHGAP33* | Rho GTPase activating protein 33 | 2.5 |
| A_33_P3301291 | *FOXB2* | lnc-LRGUK-1, lincRNA | 2.4 |
| A_21_P0001923 | *ENST00000450667* | osteoclast associated, immunoglobulin-like receptor | 2.4 |
| A_21_P0012041 | *AOX2P* | Unknown | 2.4 |
| A_33_P3346966 | *SPAG16* | sperm associated antigen 16 | 2.4 |
| A_33_P3237899 | *CHN2* | chimerin 2 | 2.4 |
| A_24_P101812 | *ENST00000548943* | coiled-coil domain containing 78 | 2.4 |
| A_33_P3275826 | *A_33_P3275826* | Unknown | 2.4 |
| A_33_P3405897 | *PLEKHM1* | pleckstrin homology domain containing, family M member 1 | 2.4 |
| A_33_P3374893 | *LINC00487* | long intergenic non-protein coding RNA 487 | 2.4 |
| A_21_P0006228 | *lnc-STXBP1-1* | mRNA for FLJ00398 protein | 2.4 |
| A_33_P3246593 | *NUGGC* | nuclear GTPase, germinal center associated | 2.3 |
| A_23_P154771 | *DUSP15* | dual specificity phosphatase 15 | 2.3 |
| A_33_P3354975 | *MTMR9LP* | myotubularin related protein 9-like | 2.3 |
| A_21_P0013043 | *XLOC_l2_012748* | engrailed homeobox 2 | 2.3 |
| A_19_P00804093 | *CCDC149* | sorbin and SH3 domain containing 3 | 2.3 |
| A_23_P31721 | *E2F5* | E2F transcription factor 5, p130-binding | 2.3 |
| A_33_P3331687 | *GPSM1* | ref\|Homo sapiens G-protein signaling modulator 1 (GPSM1), transcript variant 1, mRNA [NM_001145638] | 2.3 |
| A_21_P0014730 | *lnc-C7orf23-1* | LOC101927420 | 2.3 |
| A_33_P3269069 | *HS2ST1* | heparan sulfate 2-O-sulfotransferase 1 | 2.3 |
| A_33_P3269631 | *NAALADL2* | Unknown | 2.3 |
| A_32_P34444 | *FHOD3* | formin homology 2 domain containing 3 | 2.3 |
| A_33_P3275741 | FCN3 | arginine vasopressin | 2.3 |
| A_21_P0010216 | ENST00000423967 | cDNA clone IMAGp971I1355 ; IMAGE:824994 5' | 2.2 |
| A_23_P111995 | LOXL2 | lysyl oxidase-like 2 | 2.2 |
| A_23_P133956 | KIFC1 | kinesin family member C1 | 2.2 |
| A_23_P109488 | PIK3IP1 | basic helix-loop-helix family, member e23 | 2.2 |
| A_23_P130735 | SLC6A16 | solute carrier family 6, member 16 | 2.2 |
| A_33_P3420259 | RNA28S5 | RNA, 28S ribosomal 5 | 2.2 |
| A_23_P207201 | CD79B | CD79b molecule, immunoglobulin-associated beta | 2.2 |
| A_21_P0007502 | LINC00173 | long intergenic non-protein coding RNA 173 | 2.2 |
| A_21_P0001440 | lnc-NTPCR-1 | mex-3 RNA binding family member D | 2.2 |
| A_23_P69326 | CADPS | Ca++-dependent secretion activator | 2.2 |
| A_23_P129157 | NEIL1 | nei endonuclease VIII-like 1 | 2.1 |
| A_21_P0000190 | AFF3 | AF4/FMR2 family, member 3 | 2.1 |
| A_21_P0002853 | ENST00000469806 | MATN1 antisense RNA 1 | 2.1 |
| A_21_P0006615 | ENST00000604086 | Unknown | 2.1 |
| A_33_P3221253 | LPP | LIM domain containing preferred translocation partner in lipoma | 2.1 |
| A_23_P47709 | FOLR2 | folate receptor 2 | 2.1 |
| A_23_P136460 | FAM13B | family with sequence similarity 13, member B | 2.1 |
| A_33_P3362601 | A_33_P3362601 | cDNA FLJ35472 fis, clone SMINT2007062 | 2.1 |
| A_21_P0003200 | lnc-BCL6-3 | lnc-BCL6-3, lincRNA | 2.1 |
| A_23_P431252 | KBTBD8 | kelch repeat and BTB domain containing 8 | 2.1 |
| A_23_P79251 | EHD3 | EH-domain containing 3 | 2.1 |
| A_23_P343900 | ENST00000450948 | immunoglobulin heavy variable 5-78 | 2.1 |
| A_24_P288915 | ENST00000445752 | coiled-coil domain containing 144B | 2.1 |
| A_23_P142294 | ETHE1 | ethylmalonic encephalopathy 1 | 2.1 |
| A_32_P424761 | MYRFL | myelin regulatory factor-like | 2.1 |
| A_21_P0002916 | ENST00000423643 | Q940D6_ARATH AT4g19390/T5K18_170 | 2.1 |
| A_33_P3213962 | OR13A1 | olfactory receptor, family 13, subfamily A, member 1 | 2.1 |
| A_23_P29594 | RPL39L | ribosomal protein L39-like | 2.1 |
| A_23_P88893 | DEF8 | differentially expressed in FDCP 8 homolog | 2.1 |
| A_24_P398810 | EIF5 | eukaryotic translation initiation factor 5 | 2.1 |
| A_33_P3370515 | A_33_P3370515 | Unknown | 2.1 |
| A_24_P406060 | RNF144B | ring finger protein 144B | 2.1 |
| A_32_P86180 | LINC00577 | long intergenic non-protein coding RNA 577 | 2.0 |
| A_23_P119196 | KLF2 | Kruppel-like factor 2 | 2.0 |
| A_32_P41553 | TSACC | TSSK6 activating co-chaperone | 2.0 |
| A_23_P211136 | BRWD1 | bromodomain and WD repeat domain containing 1 | 2.0 |
| A_23_P47282 | ST14 | suppression of tumorigenicity 14 | 2.0 |
| A_23_P26629 | PYCARD | PYD and CARD domain containing | 2.0 |
| A_33_P3334448 | SNORA62 | small nucleolar RNA, H/ACA box 62 | 2.0 |
| A_23_P99853 | FAM214A | family with sequence similarity 214, member | 2.0 |
| A_23_P202034 | GUCY2GP | chromosome 19 open reading frame 26 | 2.0 |
